# Comparative Effectiveness of Aquatic Versus Land-Based Exercise on Pain, Physical Function, and Quality of Life in People with Knee Osteoarthritis: An Updated Systematic Review of Randomised Controlled Trials

**DOI:** 10.3390/healthcare14203388

**Published:** 2026-10-11

**Authors:** Mariana Amaral, Beatriz Correia, Mariana Oliveira, Beatriz Santos, Ricardo Maia Ferreira, Rui Soles Gonçalves

**Affiliations:** 1 Coimbra Health School, Polytechnic University of Coimbra, Rua 5 de Outubro, 3045-043 Coimbra, Portugal; a2022147263@estesc.ipc.pt (M.A.); a2022141961@estesc.ipc.pt (B.C.); a2022133750@estesc.ipc.pt (M.O.); a2022147523@estesc.ipc.pt (B.S.); rferreira@ipmaia.pt (R.M.F.); 2 Social Sciences, Education and Sport Department, Polytechnic Institute of Maia, N2i, 4475-690 Maia, Portugal; 3 Sport Physical Activity and Health Research & Innovation Center (SPRINT), 4960-320 Melgaço, Portugal; 4 H&TR-Health & Technology Research Center, Coimbra Health School, Polytechnic University of Coimbra, Rua 5 de Outubro, 3045-043 Coimbra, Portugal

**Keywords:** knee osteoarthritis, aquatic exercise, land-based exercise, pain, physical function, quality of life

## Abstract

**Background/Objectives:** Knee osteoarthritis (OA) causes pain, physical function limitations, and reduced quality of life. This updated systematic review compared the effectiveness of aquatic exercise (AE) versus land-based exercise (LBE) on these outcomes in adults with knee OA. **Methods:** Randomised controlled trials (RCTs) published between September 2018 and July 2025 were included if they compared supervised AE with LBE. PubMed, Web of Science, Scopus, and PEDro were searched. Methodological quality was assessed using the PEDro scale, and the certainty of evidence was evaluated using GRADE. Owing to substantial clinical and methodological heterogeneity in participant characteristics, intervention content and dose, outcome measures, and reporting methods, quantitative pooling was not considered clinically meaningful; therefore, a structured synthesis without meta-analysis was conducted. **Results:** Nine RCTs enrolling 700 participants were included, of whom 661 contributed directly to the AE versus LBE comparison. Between-group effects varied across outcomes and studies. Differences in pain measured using the VAS generally ranged from −1.80 to −0.32 scale units in favour of AE; however, clinical importance remained uncertain because applicable thresholds were not available for all measures and the confidence interval around the largest estimate included effects below the contextual benchmark. Evidence for physical function and quality of life was inconsistent, and several confidence intervals included no between-group effect or were wide. Although selected outcomes favoured AE, the observed differences were generally small or imprecise, and their clinical importance was frequently uncertain. The certainty of evidence ranged from very low to high and was predominantly low or very low. **Conclusions:** The evidence included in this update does not establish consistent clinically important superiority of AE over LBE for people with knee OA. Although some between-group estimates favoured AE, most effects were small or imprecise, and confidence in the findings was limited. Exercise modality should therefore be selected according to individual characteristics, preferences, accessibility, and clinical context. Further well-designed multicentre trials with standardised outcome reporting, longer-term follow-up, and comprehensive adherence assessment are needed.

## 1. Introduction

Osteoarthritis (OA) is the most prevalent musculoskeletal disorder worldwide and a leading cause of pain, disability, and reduced quality of life. Its aetiology is multifactorial, involving interactions between systemic factors, including ageing, obesity, and metabolic comorbidities, and mechanical factors, such as joint injury and biomechanical stress [1,2,3]. The knee is the most commonly affected joint and a major contributor to the increasing global burden of OA, which is expected to continue rising with population ageing and the growing prevalence of obesity [3,4,5,6,7].

Knee OA is characterised by progressive degeneration of joint tissues, including cartilage, ligaments, tendons, and the joint capsule. This structural deterioration contributes to pain, joint stiffness, muscle weakness, impaired proprioception, and functional decline, collectively resulting in substantial reductions in quality of life [8,9,10]. While no treatment can reverse the structural damage of OA, therapeutic strategies focus on alleviating pain and other symptoms, preserving or improving joint function, enhancing overall physical capacity, and maintaining quality of life, typically through individualised, multimodal management plans combining rehabilitation, exercise, education, weight management, and adjunctive interventions [11,12,13].

Physiotherapy, particularly exercise-based interventions such as muscle-strengthening and aerobic programmes, is recognised as a cornerstone of conservative management for knee OA, due to its proven effectiveness in reducing pain, improving physical function, and demonstrating cost-effectiveness compared with usual care [14,15,16,17]. International and national clinical guidelines consistently recommend physiotherapy-led exercise interventions, including both land-based and aquatic modalities, as first-line approaches for symptom management and functional improvement in people with knee OA [11,18,19]. Recent evidence from systematic and umbrella reviews confirms the effectiveness of exercise-based interventions, including resistance and aquatic modalities, in improving pain, function, and quality of life in patients with knee OA [20,21,22].

LBE programmes, especially resistance and strengthening exercises targeting periarticular musculature, are fundamental for improving physical function and functional capacity and for supporting joint stability in individuals with knee OA [23,24]. These interventions have consistently demonstrated reductions in pain and improvements in muscle strength, physical function, and activities of daily living [24,25,26]. However, certain patient populations, especially those with higher body mass index (BMI), pronounced pain, or functional limitations, may face substantial barriers to adherence to LBE programmes, as increased mechanical loading of the knee joint may aggravate symptoms and limit exercise tolerance [27,28,29].

AE consists of structured strength, aerobic, flexibility, balance, and functional exercises performed in water, commonly at thermoneutral or warm temperatures. Several properties of the aquatic environment provide a physiological rationale for its potential advantages over LBE. Buoyancy reduces the proportion of body weight transmitted through the lower limbs and may therefore decrease knee-joint loading and pain during weight-bearing movement. Water viscosity provides accommodating multidirectional resistance, allowing muscle activation and progressive exercise without the impact associated with some land-based activities. Hydrostatic pressure may enhance peripheral circulation, assist fluid redistribution, and provide continuous sensory input that may support proprioception and postural control. In addition, warm water may reduce pain and stiffness, facilitate muscle relaxation, and increase soft-tissue extensibility, thereby allowing a greater range of motion and improved exercise tolerance [30,31,32,33]. These mechanisms may enable individuals with pronounced pain, obesity, or functional limitations to exercise with greater comfort and engagement than on land [32,34,35]. However, these mechanisms provide a physiological rationale for potential benefit rather than establishing the clinical superiority of AE over LBE.

Despite the apparent benefits of both modalities, comparative evidence regarding their relative effectiveness remains inconclusive. In their 2018 systematic review and meta-analysis, Dong et al. [36] synthesised eight randomised controlled trials (RCTs) evaluating AE versus LBE for knee OA. The review found no statistically significant differences between the two interventions in reducing pain, improving physical function, or enhancing quality of life in both the short and long term; however, small sample sizes, heterogeneity of exercise protocols, and limited methodological quality constrained the strength of the evidence [36].

Since the publication of that review, several additional RCTs have been conducted, providing new insights into the comparative effects of AE and LBE in contemporary patient populations. Given the ongoing uncertainty regarding optimal exercise prescription for knee OA and the expansion of the evidence base, an updated systematic review is warranted. The present updated systematic review aimed to synthesise RCT evidence published between 25 September 2018 and 31 July 2025 comparing AE with LBE for pain, physical function, and quality of life in individuals with knee OA.

## 2. Materials and Methods

### 2.1. Data Sources and Search Strategy

This systematic review was conducted following established methodological standards for evidence synthesis in health sciences [37] and adhered to the PRISMA 2020 guidelines (Preferred Reporting Items for Systematic Reviews and Meta-Analyses) [38,39], as well as the SWiM (Synthesis Without Meta-analysis) reporting guidelines [40]; the full PRISMA checklist is available in Tables S1 and S2. The review methodology was defined a priori, and all procedures were applied consistently throughout the review process. The protocol was registered in the PROSPERO database (CRD420251143959).

Electronic database searches were conducted in PubMed, Web of Science, Scopus, and PEDro from inception (earliest available records) to 31 July 2025. In addition, a manual search of reference lists of the RCTs included in the current review was performed to identify potentially relevant studies not captured by the electronic searches [41].

In PubMed, the search strategy combined Medical Subject Headings (MeSH) and free-text terms corresponding to the PICO components of the review question, with searches conducted in both MeSH and title/abstract fields. In Web of Science and Scopus, only free-text terms were used, with searches conducted in the TS field (Web of Science) and TITLE-ABS-KEY fields (Scopus). In the PEDro database, searches were performed in titles and abstracts, applying the internal specifications “clinical trial”, “lower leg or knee”, and “match all search terms (AND)”. The search strategy included key terms, including but not limited to ‘Knee Osteoarthritis’, ‘Aquatic Exercise’, ‘Land-Based Exercise’, ‘Pain’, ‘Physical Function’, ‘Quality of Life’, and related synonyms. This approach ensured comprehensive coverage across all databases. The complete search strategies for each database are provided in Appendix A.

References were managed using EndNote Basic (online), where only studies published after 25 September 2018 were retained for screening, because the comprehensive systematic review by Dong et al. [36] searched the literature from database inception up to 24 September 2018, and the present review aimed to capture evidence published thereafter.

### 2.2. Research Question

The research question was formulated according to the PICOS framework (Population, Intervention, Comparison, Outcomes, and Study design). The review focused on individuals with clinically or radiologically diagnosed knee OA, irrespective of age, sex, BMI, or disease severity.

The intervention of interest was AE, delivered in a therapeutic or pool-based environment. AE programmes were defined as supervised exercise protocols and could include aerobic, strengthening, balance, proprioceptive, flexibility, or functional exercises, either as standalone interventions or as part of a structured exercise programme. The comparator intervention was LBE, consisting of supervised conventional exercise protocols performed on land. For both AE and LBE, exercise protocols were considered irrespective of exercise modality, session components, setting, frequency, session duration, total intervention length, training load, or progression strategy.

Primary outcomes of interest were pain, physical function, and quality of life, assessed using validated patient-reported outcome measures. Only RCTs were eligible; pilot studies without full methodological reporting were excluded, ensuring the highest level of evidence regarding intervention effectiveness.

### 2.3. Types of Outcome Measures

#### 2.3.1. Primary Outcomes

The primary outcomes considered in this review were pain, physical function, and quality of life. Pain was captured using the Visual Analogue Scale (VAS) [42] and disease-specific instruments, including the Western Ontario and McMaster Universities Osteoarthritis Index (WOMAC) pain subscale [43] and the Knee injury and Osteoarthritis Outcome Score (KOOS) pain subscale [44,45].

Physical function was assessed through the WOMAC physical function subscale and the KOOS subscales for function in daily living and function in sport and recreation, reflecting both basic and more demanding functional activities.

Quality of life was evaluated using validated generic instruments. The World Health Organisation Quality of Life-BREF (WHOQOL-BREF) questionnaire provides scores for the physical, mental, social, and environmental domains, as well as a total score [46]. The 12-item Short Form Health Survey (SF-12) includes the physical and mental domains [47], while the 36-item Short Form Health Survey (SF-36) covers physical functioning, role-physical, bodily pain, general health, vitality, social functioning, role-emotional, and mental health [48,49,50].

These outcome measures were predefined to provide a consistent and comprehensive assessment of the impact of knee OA and the potential effectiveness of the interventions under investigation.

#### 2.3.2. Secondary Outcomes

Secondary outcomes were compiled from the included studies and encompassed a wide range of domains, including stiffness, functional performance, cardiorespiratory capacity, balance, knee instability, range of motion, lower limb muscle strength, functional mobility, flexibility, joint position sense, muscle activity, and disease-specific severity. Unlike the primary outcomes, these measures were not predefined but rather reflect the diversity of instruments employed across the included trials, providing complementary insights into the multidimensional impact of knee OA.

Stiffness was assessed using the WOMAC stiffness subscale. Functional performance was evaluated through the 6-Minute Walk Test [51] and stair tests [52]. Cardiorespiratory capacity was measured using maximum oxygen consumption [53], anaerobic threshold [54], and exertion-based scales such as the Borg scale and Borg CR10 scale [55,56].

Balance was assessed with the Berg Balance Scale [57], while knee instability was evaluated via Felson’s questionnaire [58]. Range of motion was measured using a goniometer, bubble inclinometer, or 2D video-based kinematics [59].

Lower limb muscle strength was quantified using manual muscle testing [60], 1RM leg press [61], digital dynamometry for knee extensor assessment [62], and the Five Times Sit-to-Stand Test (5xSTS) [63]. Functional mobility and balance were assessed with the Timed Up-and-Go Test [64], and walking speed was measured using the 40 m fast-paced walk test [65]. Joint position sense was evaluated with a goniometer [59].

Muscle activity outcomes were captured using electromyography, examining individual muscles such as the rectus femoris, vastus lateralis, biceps femoris, and semi-membranosus, as well as overall muscle activity and extensor/flexor co-contraction patterns [66].

Flexibility of the hamstrings and hip was assessed using the Active Knee Extension Test [67] and the Modified Thomas Test [67], respectively. Quadriceps amyotrophy was evaluated by measuring the difference in muscle circumference between quadriceps [68]. Finally, disease-specific severity was quantified using the Lequesne Index [69].

### 2.4. Study Selection and Data Extraction

EndNote Basic (online) was used to manage references and remove duplicate records. Study selection was conducted in two sequential stages. First, titles and abstracts were screened to identify potentially eligible studies. Subsequently, full-text articles were assessed for inclusion. Both stages were performed independently by two reviewers. Disagreements were resolved in a consensus meeting with a third reviewer, ensuring that all decisions were discussed collectively and made transparently. All included studies were published in languages understood by the reviewers (English and Portuguese); therefore, no translation tools were required.

Data extraction was independently performed by two reviewers using the full text of each included study. Data were entered directly into structured tables developed specifically for this review, which served as the data extraction framework. The predefined data items included: study identification (authors, year, and country); participant characteristics, including age, sex distribution, group-specific sample sizes, BMI, diagnostic criteria, OA laterality, and Kellgren–Lawrence grade; intervention characteristics, including exercise modality, session components, setting, frequency, session duration, total intervention length, training load, and progression; and outcome information, including outcome domain, measurement instrument, group-specific numerical data, and the main study findings. Differences in extraction decisions were resolved through discussion and, when necessary, consultation with a third reviewer.

Post-intervention means, standard deviations, and group-specific sample sizes were extracted for each continuous outcome to calculate unadjusted post-intervention between-group mean differences and 95% confidence intervals (CIs). Baseline values were extracted to describe group characteristics and permit assessment of potential baseline imbalance. Baseline-adjusted estimates were preferentially sought but were not consistently reported by the included studies. Within-group statistical significance was not used to estimate comparative intervention effects. For multi-arm trials, only data from the AE and LBE groups were used to calculate the direct between-group comparisons; participants allocated to additional control groups were not included in these calculations.

When data were missing or unclear, the information was recorded as “not reported.” In cases of missing or ambiguous data, the authors of seven original RCTs were contacted; however, no responses were obtained. Importantly, no studies were excluded on this basis, and only the missing or ambiguous data were omitted from the analyses.

### 2.5. Eligibility Criteria

#### 2.5.1. Inclusion Criteria

Studies were included if they met all of the following: (1) RCTs meeting the predefined population, intervention, comparator, and outcome criteria; (2) Published in peer-reviewed journals between 25 September 2018 and 31 July 2025; (3) Full text available in English or Portuguese; (4) Participants diagnosed with knee OA based on clinical and/or radiological criteria; (5) Experimental group undertaking AE; (6) Control group undertaking LBE; (7) Detailed description of both exercise interventions provided; and (8) Assessment of at least one clinically relevant outcome: pain, physical function, or quality of life. Outcome measures: Pain had to be assessed using the VAS, WOMAC, and/or KOOS; physical function via WOMAC and/or KOOS; and quality of life via SF-12, SF-36, and/or WHOQOL-BREF.

#### 2.5.2. Exclusion Criteria

Studies were excluded if they met any of the following: (1) Study designs other than RCTs (e.g., systematic reviews, cohort or case–control studies, case reports or series, editorials, expert opinions); (2) Grey literature; (3) Retracted or withdrawn publications; (4) Animal studies; (5) Participants without a confirmed diagnosis of knee OA or with prior total knee replacement; (6) Presence of comorbidities (musculoskeletal, neurological, oncological) likely to affect pain, function, or quality of life outcomes; or (7) Interventions solely pharmacological or surgical, or concomitant treatments likely to influence physiotherapy outcomes.

### 2.6. Methodological Quality Assessment

The methodological quality of the included studies was appraised using the Portuguese-adapted version of the PEDro scale [70], an established instrument for evaluating the design and reporting of RCTs in physiotherapy [71,72,73,74,75], and following procedures previously applied by the authors in related systematic reviews [76,77]. The scale comprises eleven criteria, of which ten contribute to the total score. The first item, regarding eligibility criteria and external validity, is documented but not scored. The remaining items cover key domains, including random sequence generation, allocation concealment, baseline comparability, blinding of participants, therapists, and outcome assessors, completeness of outcome data (≥85% follow-up), application of intention-to-treat principles, reporting of between-group comparisons, and presentation of point estimates with measures of variability. Total scores range from 0 to 10, with 9–10 indicating excellent quality, 6–8 good, 4–5 fair, and ≤3 poor. Quality assessment was conducted independently by two reviewers. Any disagreements were resolved by discussion with a third reviewer. This process facilitated the identification of potential methodological limitations and supported a rigorous and transparent interpretation of the findings.

### 2.7. Data Synthesis and Analysis

Quantitative data from the included studies were systematically extracted and tabulated to allow structured comparisons between AE and LBE interventions, including pre- and post-intervention values and between-group mean differences with 95% CIs. When standard deviations were not reported but standard errors were available, standard deviations were estimated using the formula SD = SE × √n. Data presented only in graphical form were digitised using the WebPlotDigitizer online tool, version 5 (https://automeris.io/WebPlotDigitizer/), accessed on 15 December 2025 [78]. In cases where studies reported median, minimum, and maximum values instead of mean and standard deviation, sample means and standard deviations were estimated using well-established methods described in the literature [79,80,81,82], as implemented via an online calculator available at https://www.math.hkbu.edu.hk/~tongt/papers/median2mean.html (accessed on 15 December 2025). Between-group mean differences and their corresponding 95% CIs were calculated using standard formulae [41] via the online calculator provided at https://www.statskingdom.com/difference-confidence-interval-calculator.html (accessed on 15 December 2025).

The appropriateness of meta-analysis was assessed separately for each outcome domain and measurement instrument. Although clinical heterogeneity alone does not necessarily preclude the use of a random-effects model, quantitative pooling was not undertaken because most outcome-instrument combinations were informed by few studies and the trials differed substantially in intervention content and dose, participant characteristics, scale ranges, reporting methods, and the treatment of bilateral or multiple correlated measurements. Several studies also involved aquatic and land-based programmes that differed in more than exercise environment, limiting the clinical interpretability of a pooled estimate. Intervention comparability was therefore assessed descriptively using the information reported in the included studies. Programmes were classified into three categories according to intervention comparability. They were considered closely matched when exercise content, frequency, session duration, and progression were broadly comparable, with the exercise environment representing the principal difference. Programmes were considered partially matched when they shared major exercise components but differed meaningfully in content or dose. They were classified as fundamentally different when distinct exercise modalities or multimodal programmes prevented the exercise environment from being isolated as the principal contrast. A structured synthesis without meta-analysis was therefore conducted in accordance with the SWiM reporting guideline [40]. Studies were grouped by outcome domain and measurement instrument. The principal quantitative information used in the synthesis was the unadjusted post-intervention between-group mean difference and corresponding 95% CI. These estimates were interpreted descriptively rather than as baseline-adjusted treatment effects. Adjusted estimates could not be reconstructed because they were not consistently reported and individual participant data were unavailable. Between-group differences in change from baseline were not calculated because the standard deviations of the changes and the within-participant baseline-to-follow-up correlations were not consistently reported. Estimating change-score variances would therefore have required imputing unreported correlations and introducing additional assumptions. Furthermore, change-score calculations would not adjust for imbalances in age, BMI, disease severity, or other prognostic characteristics. The synthesis considered the magnitude, direction, and precision of the estimates, consistency across studies and instruments, sample size, methodological quality, and clinical importance when available. When a potentially relevant threshold was available, its applicability was assessed in relation to the outcome instrument, scale range, population, follow-up context, and whether it represented within-person change or a between-group difference. Statistical significance was not used as the basis for an overall vote count. Multiple subscales, individual muscle measurements, and bilateral outcomes reported within the same study were treated as correlated results from a single participant sample and were not interpreted as independent units of evidence. Formal statistical heterogeneity and sensitivity analyses were not performed because quantitative pooling was not undertaken and most outcome categories contained too few comparable studies to support reliable analyses [41,83].

### 2.8. Certainty of Evidence Assessment

The certainty of evidence for each outcome was assessed using the GRADE (Grading of Recommendations Assessment, Development and Evaluation) approach [84,85,86]. GRADE evaluates five domains: risk of bias, inconsistency, indirectness, imprecision, and publication bias. These domains were selected because they comprehensively capture the main sources of uncertainty in the evidence and are standard within the GRADE framework. Risk of bias for the GRADE assessment was evaluated at the outcome level for each study–outcome pair, using the Cochrane Handbook bias domains. The individual PEDro design items informed these judgements: randomization process (items #2–3), deviations from intended interventions (items #5–6), missing outcome data (items #8–9), and measurement of the outcome (item #7). The influence of absent participant and therapist blinding was weighted according to outcome type, being considered high risk for self-reported outcomes and lower risk for performance-based outcomes measured by blinded assessors. Selection of the reported result could not be assessed from design items alone. Judgements were made independently by two reviewers, with disagreements resolved by a third. Due to clinical and methodological heterogeneity across the included studies, meta-analysis was not performed; therefore, the certainty of evidence was assessed through qualitative synthesis for each outcome [87]. Certainty was assessed across studies rather than individual studies and categorised into four levels: high, moderate, low, or very low. The GRADE assessment was prepared using GRADEpro GDT (https://www.gradepro.org/), an online platform for structured evidence assessment.

## 3. Results

### 3.1. Study Selection Process

The systematic process of study selection, encompassing the phases of identification, screening, and inclusion, is detailed in the PRISMA 2020 flow diagram (Figure 1). Initially, 932 records were identified through electronic database searches, including 146 from PubMed, 504 from Web of Science, 222 from Scopus, and 60 from PEDro, with an additional 222 records retrieved via manual citation searching, yielding a total of 1154 records. Of note, 220 of the 222 citation-search records were duplicates of studies already identified through the electronic database searches.

From the records obtained through the electronic databases, 175 duplicates were removed, and a further 464 records were excluded based on publication date criteria, leaving 293 records for title and abstract screening. Following this screening, 281 records were excluded for not meeting the predefined eligibility criteria. Twelve reports were sought for full-text retrieval, of which two were unavailable. Additionally, two reports identified through manual citation searching underwent full-text assessment.

In total, 12 RCTs were assessed for eligibility. Three studies were subsequently excluded: two because the control group did not perform LBE, and one because the study population did not consist exclusively of individuals with knee OA. Consequently, nine RCTs [88,89,90,91,92,93,94,95,96] were included in the final systematic review.

### 3.2. Methodological Quality of Studies

The methodological quality of the included RCTs, assessed using the PEDro scale [70,71,72,73,74,75], ranged from 4 to 8 points out of a maximum of 10. All studies met the criteria for random allocation (item #2), between-group statistical comparisons (item #10), and reporting of point estimates with measures of variability (item #11) [88,89,90,91,92,93,94,95,96].

None of the studies implemented participant blinding (item #5) or therapist blinding (item #6). Other methodological criteria, namely concealed allocation (item #3), baseline group similarity (item #4), assessor blinding (item #7), availability of outcome data for more than 85% of participants (item #8), and intention-to-treat analysis (item #9), were inconsistently reported across studies [88,89,90,91,92,93,94,95,96].

Three studies were classified as having fair methodological quality (PEDro scores 4–5/10: Slouma et al. [95], Tamin & Loekito [96], and Krishnan et al. [92]), while the remaining six studies were rated as having good methodological quality (scores 7–8/10) [88,89,90,91,93,94]. Detailed PEDro item scores for each study are presented in Table 1.

### 3.3. Study Characteristics

The nine included RCTs enrolled a total of 700 participants, of whom 661 were allocated to the AE and LBE groups and contributed directly to the comparative evidence of interest. The remaining 39 participants were allocated to third control groups in three trials and were not included in the AE versus LBE between-group comparisons. Considerable variability was observed in sample size, sex distribution, age, BMI, diagnostic criteria, OA laterality, and disease severity across studies (Table 2). Sample sizes per group varied substantially, ranging from 8 [88] to 145 participants [90]. The proportion of female participants ranged from 40% [92] to 100% [88,89,93,94], and mean age varied from 49.47 [92] to 68 years [88]. BMI values ranged from 23.1 [89] to 34.41 kg/m^2^ [92]. OA was diagnosed according to the ACR criteria in three studies [88,89,95], whereas the remaining studies used clinical diagnosis by physicians or did not specify the diagnostic criteria [90,91,92,93,94,96]. Two studies explicitly included participants with unilateral or bilateral OA [88,95], three studies included only bilateral OA [89,90,94], and in the remaining four studies OA laterality was not reported [91,92,93,96]. Kellgren–Lawrence grades spanned from I to IV across studies, although several trials applied more restrictive inclusion criteria, such as limiting participation to intermediate disease stages (e.g., grades II–III) [91,96].

Intervention duration ranged from 6 [91] to 12 weeks [88], with exercise sessions delivered two or three times per week and lasting between 40 [88,90] and 90 min [89], depending on the study (Table 3). Follow-up assessments beyond the immediate post-intervention period were reported in only one study, which evaluated quality-of-life outcomes six months after completion [91].

AE interventions varied in structure but generally included combinations of aerobic, strengthening, balance, proprioceptive, and flexibility exercises performed in warm or thermoneutral water under supervision. Session components typically comprised warm-up, main exercise phases, and cool-down, with water depth and temperature specified in most protocols. Training load and progression were reported in the majority of aquatic interventions and were achieved through increases in exercise intensity, repetitions, sets, duration, movement complexity, or task difficulty, although some studies [93,95] applied fixed exercise programmes without explicit progression.

LBE interventions were similarly heterogeneous and included strengthening, functional, balance, proprioceptive, aerobic, and flexibility exercises, primarily targeting the lower limbs. These programmes were conducted in gym or clinical settings and were supervised across studies [88,89,90,91,92,93,94,95,96]. Progression strategies, when reported, involved increases in repetitions, sets, resistance, duration, or exercise complexity, while a small number of studies [93,95] described land-based protocols with fixed training volumes and no systematic progression. Based on the descriptive assessment of intervention comparability, four studies were classified as closely matched because the aquatic and land-based programmes had broadly comparable exercise content and dose [88,90,91,93]. Two studies were classified as partially matched because the programmes shared major exercise components but differed in session duration, content, or delivery [94,95]. Three studies were classified as fundamentally different because AE was compared with a distinct land-based modality, including TRX, TheraBand exercise, or cycling combined with strengthening [89,92,96]. These latter comparisons do not isolate exercise environment as the principal difference between groups.

### 3.4. Primary Outcomes

Unadjusted post-intervention between-group estimates for the primary outcomes varied in magnitude, direction, and precision across studies and measurement instruments (Table 4).

For pain measured using the VAS, statistically significant between-group mean differences favouring AE ranged from −0.32 to −1.80 points [90,94,95]. Most of these estimates were smaller than one point, with relatively narrow 95% CIs that excluded zero, indicating relatively precise but generally small differences between interventions. The largest estimate was −1.80 points (95% CI −2.82 to −0.78) [95], although the confidence interval extended from a relatively small to a potentially clinically important effect. Other pain estimates were imprecise or compatible with no between-group effect. These included VAS activity in Assar et al. [89] (−0.30, 95% CI −1.94 to 1.34), unspecified VAS pain in Krishnan et al. [92] (−0.13, 95% CI −0.62 to 0.36), WOMAC pain in Arrieiro et al. [88] (−27.98, 95% CI −154.17 to 98.21) and Khruakhorn and Chiwarakranon [91] (−0.47, 95% CI −6.16 to 5.22), and KOOS pain in Pezeshk et al. [93] (2.82, 95% CI −15.88 to 21.52). The width of some of these intervals indicates considerable uncertainty and does not permit firm conclusions regarding the direction or magnitude of the comparative effect.

For physical function, the between-group estimates were also inconsistent. The unadjusted WOMAC physical function estimate reported by Slouma et al. [95] was numerically in favour of AE (−22.70, 95% CI −28.64 to −16.76). However, the groups differed at baseline, and the reported post-intervention estimate was not adjusted for that imbalance. Therefore, its magnitude should not be interpreted as an adjusted treatment effect. In contrast, the estimates from Arrieiro et al. [88] (−53.97, 95% CI −392.34 to 284.40) and Khruakhorn and Chiwarakranon [91] (−4.11, 95% CI −21.13 to 12.91) had confidence intervals that included zero, with the particularly wide interval in Arrieiro et al. [88] indicating substantial imprecision. WOMAC total score estimates ranged from −4.11 to −31.40 points. Three estimates favoured AE and had confidence intervals excluding zero [90,94,95], whereas the estimate from Khruakhorn and Chiwarakranon [91] was imprecise and compatible with effects in either direction (−4.11, 95% CI −28.72 to 20.50). Differences in reported WOMAC scale ranges limit direct comparisons of effect magnitude across studies.

Quality-of-life estimates varied in both direction and precision. For the WHOQOL-BREF domains and total score [91], all confidence intervals included zero, indicating no clear evidence of a between-group difference. In Slouma et al. [95], the unadjusted post-intervention SF-12 estimates were numerically in favour of AE for the physical domain (10.70, 95% CI 7.48 to 13.92) and mental domain (5.80, 95% CI 0.69 to 10.91). However, baseline SF-12 scores differed between groups, particularly for the physical domain, and these post-intervention differences should therefore not be interpreted as baseline-adjusted treatment effects. Shinde and Jain [94] reported estimates favouring AE across the SF-36 domains, generally ranging from 2.76 to 5.88 points, with narrow confidence intervals. In contrast, Tamin and Loekito [96] reported estimates favouring LBE for physical functioning (−21.94, 95% CI −35.90 to −7.98), role-physical (−26.10, 95% CI −46.23 to −5.97), and social functioning (−17.15, 95% CI −27.21 to −7.09), although these estimates were derived from a study with only 33 participants. Other SF-36 estimates had confidence intervals that included zero and were therefore compatible with no between-group effect. Overall, the quality-of-life evidence was inconsistent across studies and instruments, preventing a firm conclusion regarding the comparative effect of AE and LBE.

### 3.5. Secondary Outcomes

Unadjusted post-intervention between-group estimates for the secondary outcomes were heterogeneous in magnitude, direction, and precision across studies and outcome domains (Table 5). As with the primary outcomes, these estimates were interpreted descriptively because baseline-adjusted treatment effects were not consistently available. The secondary outcomes included stiffness and disease-specific severity, functional performance, lower-limb muscle strength, joint flexibility and position sense, muscle activity, range of motion, balance, and cardiorespiratory measures [88,89,90,91,92,93,94,95,96]. Because several studies reported multiple correlated subscales, individual muscle measurements, or bilateral outcomes from the same participants, these estimates were interpreted as related results within each study rather than as independent evidence. The synthesis therefore focused on study-level patterns, effect magnitude, and 95% CIs rather than on the number of statistically significant comparisons.

For stiffness measured using the WOMAC subscale, three studies reported estimates with 95% CIs that included zero [88,89,91]. One study reported an estimate favouring AE (−2.50, 95% CI −3.51 to −1.49) [95]. Disease-specific severity, assessed using the Lequesne index, was reported by the same study and also favoured AE (−5.50, 95% CI −7.30 to −3.70) [95]. Because both estimates were derived from a single study and an applicable threshold for clinical importance was not established, they do not demonstrate a consistent advantage across the evidence base, and their clinical importance remains uncertain.

Functional performance estimates varied across studies and instruments. For the 6 min walk test, one small study reported an imprecise estimate compatible with effects in either direction (−13.53, 95% CI −75.13 to 48.07) [88], whereas another small study reported an estimate favouring AE (33.40 m, 95% CI 9.12 to 57.68) [92]. For the Timed Up and Go test, one study reported an estimate favouring AE (−1.75 s, 95% CI −2.34 to −1.16) [90], while the estimate from another study included zero (−0.25 s, 95% CI −3.06 to 2.56) [91]. An estimate favouring AE was also reported for the 40 m fast-paced walk test (−2.84 s, 95% CI −3.45 to −2.23), but this outcome was reported by a single study [90]. These results do not demonstrate a consistent pattern across the available functional performance measures.

Lower-limb muscle-strength estimates differed according to the assessment method. One study reported an estimate favouring AE for the 1RM leg press (7.35 kg, 95% CI 4.97 to 9.73) [90]. Another study reported estimates favouring AE across 12 manual muscle tests, with mean differences ranging from 0.26 to 0.96 points [94]. These 12 measurements were obtained from the same participant sample and represent correlated muscle-specific results rather than 12 independent confirmations of an effect. In contrast, the estimate for knee-extensor strength measured using dynamometry included zero (−0.50 kg, 95% CI −3.55 to 2.55) [89]. Consequently, the available evidence suggests possible study-specific differences but does not establish a consistent effect across strength-assessment methods.

Estimates for flexibility, range of motion, and joint position sense were also derived predominantly from individual studies reporting multiple related measurements. One study reported estimates favouring AE for hamstring and hip flexibility, including bilateral measurements from the same participants [94], and another reported a goniometric range-of-motion estimate favouring AE (11.0°, 95% CI 5.45 to 16.55) [95]. In contrast, a study using a bubble inclinometer reported an estimate favouring LBE for knee range of motion (−7.50°, 95% CI −13.46 to −1.54) [89], while a study using two-dimensional video-based kinematics reported an estimate close to no between-group difference (−0.14°, 95% CI −5.41 to 5.13) [93]. Bilateral joint-position-sense estimates favoured AE in one study [90], but the two knee measurements were obtained from the same participant sample and should not be interpreted as independent evidence. The direction and magnitude of these results therefore varied by study and assessment method.

Cardiorespiratory estimates were reported by one study [88]. The 95% CIs for maximal oxygen consumption (−0.18 mL·kg^−1^·min^−1^, 95% CI −4.58 to 4.22) and anaerobic threshold (3.50 mL·kg^−1^·min^−1^, 95% CI −1.27 to 8.27) included zero and were compatible with effects in either direction. The limited sample size and imprecision prevent firm conclusions regarding comparative cardiorespiratory effects.

### 3.6. Certainty of the Evidence

The overall certainty of the evidence for each outcome is presented in Table 4 and Table 5. Taken together, the GRADE synthesis indicates that the certainty of evidence varied across outcomes. For primary outcomes, certainty was predominantly low to very low for pain, physical function and quality of life, reflecting limitations related to risk of bias, inconsistency, and imprecision (Table 4). For functional mobility and balance, as assessed by the Timed Up and Go test, the certainty of evidence was moderate. Other secondary outcomes (stiffness and functional performance) were generally rated between low and very low due to similar limitations (Table 5). Additional outcomes informed by a single study were also assessed, with certainty ratings ranging from low to high. These ratings should be interpreted cautiously because they were based on evidence from only one study for each outcome (Table S3).

## 4. Discussion

The present updated systematic review synthesised evidence from nine RCTs published between 25 September 2018 and 31 July 2025 [88,89,90,91,92,93,94,95,96] comparing AE with LBE in individuals with knee OA. Overall, the findings varied across studies, outcomes, and measurement instruments and did not establish consistent clinically important superiority of either exercise modality. Although some estimates favoured AE, the observed differences were generally small, imprecise, or of uncertain clinical importance, and confidence in the evidence was limited.

A previous systematic review by Dong et al. [36] concluded that AE and LBE produce broadly comparable effects on pain, physical function, and quality of life in individuals with knee OA. The comparative effects varied across studies, outcomes, and measurement instruments. Although several estimates favoured AE, most differences in pain were small and several confidence intervals included no between-group effect. For the largest VAS estimate, the point estimate marginally exceeded a contextual clinical-importance benchmark, but its confidence interval included effects below that benchmark. Estimates favouring LBE for selected quality-of-life domains were derived from a single study with 33 participants [96], and should likewise be interpreted cautiously. Pain reductions observed in AE groups, particularly in VAS, were generally small to moderate [90,94,95]. For contextual interpretation, the VAS activity estimate reported by Slouma et al. [95] was compared with a published 1.75 cm threshold for the 10 cm VAS [97]. The point estimate of −1.80 cm marginally exceeded this benchmark and its 95% CI (−2.82 to −0.78) excluded no between-group difference. However, the interval also included effects smaller than 1.75 cm. Therefore, this result does not establish with confidence that the true between-group difference reached the contextual threshold. The remaining VAS estimates were smaller than this benchmark or had confidence intervals compatible with no between-group difference. Because the threshold was not prespecified and may not correspond directly to every study population, pain context, or type of between-group estimate, these comparisons should be regarded as contextual rather than definitive assessments of clinical importance. These results suggest that, although AE may confer some advantages over LBE, the magnitude of benefit varies across populations and outcome measures. The confidence intervals further indicate considerable variation in precision. While several estimates had relatively narrow intervals excluding no effect, others included zero or were sufficiently wide to remain compatible with effects in either direction. Consequently, statistical significance alone should not be interpreted as evidence of a clinically important or consistently estimated advantage of AE. The properties of the aquatic environment provide plausible mechanisms through which AE may facilitate exercise in some individuals with knee OA. Buoyancy reduces the proportion of body weight transmitted through the lower limbs, which may decrease knee-joint loading and permit painful or limited movements to be performed with greater tolerance. Water viscosity provides accommodating multidirectional resistance, allowing muscle activation throughout movement while limiting the impact associated with some land-based tasks. Hydrostatic pressure may contribute to fluid redistribution and continuous somatosensory input, potentially supporting proprioception, postural control, and movement confidence. When exercise is performed in warm water, thermal effects may also reduce pain and stiffness, promote muscle relaxation, and increase soft-tissue extensibility, thereby facilitating range of motion and exercise participation [30,31,32,33,34,35,98]. These mechanisms may be particularly relevant for individuals with pronounced pain, obesity, or weight-bearing limitations. Nevertheless, they indicate biological plausibility and the potential to improve exercise tolerance rather than demonstrating superior clinical effectiveness of AE over LBE.

Physical function outcomes, as assessed by the WOMAC physical function subscale, showed mixed results between groups. In the study by Slouma et al. [95], the unadjusted post-intervention mean difference was −22.70 points (95% CI −28.64 to −16.76). For contextual interpretation, this estimate was compared with a published 12.5-point threshold for clinically important within-person change in knee OA populations [99]. Although the point estimate and confidence interval exceeded this numerical benchmark, the threshold was not derived as a between-group difference, the WOMAC scale range was not explicitly reported in the included trial, the intervention groups differed at baseline, and the estimate was not adjusted for that imbalance. The comparison therefore does not establish that the comparative treatment effect was clinically important. Interpretation of WOMAC results in the remaining studies was further limited by the frequent absence of explicitly reported scale ranges, which prevented consistent application of clinical-importance thresholds. WOMAC total-score estimates should also be interpreted cautiously because they combine symptom and physical-function domains and were based on unadjusted post-intervention comparisons. Future studies should incorporate participant-reported engagement, enjoyment, and perceived barriers, as these factors may influence functional gains and clinical applicability.

Quality-of-life estimates were inconsistent across studies and instruments: two studies reported comparisons that tended to favour AE, particularly in the SF-12 physical and mental domains [95] and several SF-36 subscales [94], whereas one study reported estimates favouring LBE for the SF-36 physical functioning, role-physical, and social functioning subscales [96]. These findings were based on unadjusted post-intervention comparisons, and some may have been influenced by baseline differences between groups, particularly for the SF-12 physical domain in Slouma et al. [95]. These mixed findings may reflect the multidimensional nature of quality of life in individuals with knee OA, which is influenced not only by physical improvements but also by psychosocial factors such as self-efficacy, coping, and psychological distress, as well as by contextual and daily life circumstances, highlighting that the perceived impact of different exercise modalities may vary between individuals [100]. Additionally, the predominance of female participants in most studies limits the generalisability of these findings, emphasising the need for more diverse and representative samples in future trials.

Findings for secondary outcomes were heterogeneous and do not establish a consistent clinically important advantage of either exercise modality. The number of statistically significant estimates was not interpreted as a measure of the overall effect because study size varied substantially and several trials contributed multiple correlated subscales, muscle-specific outcomes, or bilateral measurements from the same participants. Selected estimates for muscle strength, flexibility, proprioception, and functional performance favoured AE, whereas one estimate for knee range of motion favoured LBE [89,90,91,92,93,94,95]. However, many of these findings were derived from individual studies, multiple related measurements within the same samples, or outcomes for which established thresholds of clinical importance were unavailable. Some studies reported between-group estimates favouring AE for lower-limb muscle strength [90,94], hip and knee flexibility [94,95], and joint position sense [90]. These findings are compatible with a possible contribution of water resistance and the continuous sensory input provided by the aquatic environment, but they do not directly demonstrate distinct neuromuscular or sensorimotor adaptations to AE [101]. Functional performance outcomes, such as timed mobility [90,91] and walking tests [90,92], showed variable effects across studies, reflecting both variations in intervention protocols and differences in participants’ baseline functional status. Stiffness, assessed via WOMAC subscales, was evaluated in four studies [88,89,91,95], with statistically significant between-group differences observed in only one trial [95]. A between-group difference favouring AE was reported for disease-specific severity assessed using the Lequesne index, but this estimate was derived from a single study and its clinical importance remains uncertain [95]. Cardiorespiratory measures generally remained comparable between interventions [88], potentially reflecting limited intervention durations or moderate exercise intensities. Nevertheless, as with the primary outcomes, the clinical importance of the statistically significant differences observed for secondary outcomes remains uncertain. These results suggest that, although AE may confer additional physiological advantages, the translation of these effects into meaningful functional gains should be interpreted with caution.

The exclusion of grey literature and the absence of searches of trial registries may have resulted in the omission of unpublished or ongoing studies and increased the risk of publication bias. Restricting eligibility to reports published in English or Portuguese may also have introduced language bias, while limiting inclusion to selected outcome instruments may have excluded RCTs assessing the same outcome constructs using other valid measures. These restrictions may have reduced the comprehensiveness and generalisability of the review findings. In addition, evidence published after 31 July 2025 falls outside the registered temporal scope of the present update and should be considered in a subsequent update. The methodological quality of the included trials also warrants careful consideration when interpreting the observed between-group effects. Overall, the studies ranged from fair to good methodological quality according to PEDro criteria and consistently satisfied core design and reporting standards, including the use of randomised group allocation, the presentation of between-group statistical analyses, and the reporting of outcome estimates accompanied by appropriate measures of variability. Nevertheless, several potential sources of bias were identified. Notably, none of the trials implemented participant or therapist blinding, a limitation that may reflect the inherent methodological challenges associated with blinding in physiotherapy interventions, given that participants are necessarily aware of the treatment received and therapists cannot be blinded to the intervention delivered [102,103]. In addition, reporting of concealed allocation, assessor blinding, intention-to-treat analyses, and completeness of outcome data was inconsistent across studies. Although the original trials frequently reported within-group pre–post changes, these changes were not interpreted as evidence of intervention effectiveness or comparative superiority because they may reflect regression to the mean, natural variation, co-interventions, or nonspecific effects. Comparative interpretation was therefore based on the available between-group estimates. A further limitation is that baseline-adjusted treatment-effect estimates were not consistently reported by the included trials. Consequently, unadjusted post-intervention between-group differences were calculated from aggregate data. These estimates may have been influenced by baseline imbalances and could not account for differences in age, BMI, disease severity, or other prognostic characteristics. Between-group differences in change from baseline were not calculated because standard deviations of the changes and within-participant baseline-to-follow-up correlations were not consistently reported. Estimating change-score variances would therefore have required imputing unreported correlations and introducing additional assumptions. The resulting estimates should be interpreted as descriptive comparisons rather than definitive causal effects. Importantly, at least some of the statistically significant findings favouring AE were derived from trials with lower PEDro scores [92,95], underscoring the need for cautious interpretation. It should be noted that the PEDro scale primarily evaluates methodological quality rather than risk of bias at the outcome level. Collectively, these methodological considerations suggest that, while the available RCTs provide valuable comparative evidence, residual limitations in internal validity may partly account for the observed between-group differences and should be considered when appraising the strength and robustness of the conclusions. In addition, inter-reviewer agreement statistics were not calculated, and the separate pre-consensus screening decisions were not retained; therefore, the level of agreement before consensus could not be quantified.

The characteristics of the included studies further contextualise the variability observed in treatment effects and help to explain the absence of consistent, clinically robust superiority of one exercise modality over the other. Across the nine RCTs, substantial heterogeneity was evident in participant profiles, including sample size, sex distribution, age, BMI, disease severity, and diagnostic criteria, all of which are known to influence responsiveness to exercise-based interventions in knee OA [104,105]. Of note, several studies included participants with BMI values within the obesity range [92,93,94,95], reflecting the real-world population of individuals with knee OA, in whom overweight and obesity are highly prevalent. The inclusion of participants with obesity reflects the clinical population affected by knee OA and was consistent with the pre-specified eligibility criteria. However, differences in BMI and other prognostic characteristics between intervention groups could not be accounted for in the unadjusted aggregate-data comparisons and therefore remain a source of uncertainty. Intervention comparability represents an additional limitation to the interpretation of the observed between-group differences. Four trials compared closely matched AE and LBE programmes [88,90,91,93], two were partially matched [94,95], and three compared fundamentally different programmes [89,92,96]. In the partially matched and fundamentally different comparisons, the groups differed not only in exercise environment but also in exercise modality, equipment, session duration, training load, progression, supervision, or other programme components. Consequently, the corresponding between-group estimates represent comparisons between complete intervention packages and cannot be attributed specifically to water immersion or to the physiological properties of the aquatic environment. Even in closely matched trials, complete isolation of the environmental effect is not possible because movement execution, resistance, equipment, and participant experience necessarily differ between aquatic and land-based settings. The mechanistic explanations discussed above should therefore be considered biologically plausible hypotheses rather than mechanisms demonstrated by the included trials.

Participant adherence to the prescribed exercise protocols was variably reported across the included RCTs. Only two studies provided detailed session-by-session attendance data, indicating high adherence rates with most participants completing the majority of supervised sessions: in one study, participants attended on average 33 of 36 planned sessions (92%) [88], while in the other, attendance was 100% in the aquatic group and 91% in the TheraBand (land-based) exercise group [92]. The remaining studies [89,90,91,93,94,95,96] reported only partial or indirect information, such as overall completion rates or dropouts, without specifying attendance at individual sessions. Overall, the available evidence suggests that participants generally followed the interventions as intended, supporting the likelihood that the observed outcomes reflect the effects of the prescribed exercise programmes. Nonetheless, incomplete reporting of adherence represents a limitation and should be considered when interpreting between-group differences, as variations in compliance may influence the observed effectiveness of AE versus LBE interventions. Adverse events were not defined as an outcome in the registered protocol and were not systematically reported across the included studies; therefore, no conclusion can be drawn regarding the comparative safety of AE and LBE.

In addition to BMI, variability in Kellgren–Lawrence grades and the frequent application of restrictive inclusion criteria targeting specific disease stages may have contributed to differential baseline symptom burden and functional capacity across studies, thereby affecting the magnitude of between-group contrasts [106]. The limited use of follow-up assessments beyond the immediate post-intervention period also restricts inferences regarding the sustainability of observed effects [107,108]. Taken together, these study-level characteristics suggest that the observed between-group differences should be interpreted within the context of diverse populations and intervention designs, rather than as evidence of uniform or generalisable advantages of one exercise modality across all individuals with knee OA. Consideration of these factors should guide stratified analyses or tailored intervention designs in future research.

Overall, the GRADE synthesis indicates that the certainty of evidence varied across outcomes. For the primary outcomes of pain, physical function and quality of life, the certainty of evidence was predominantly low to very low, reflecting limitations related to risk of bias, inconsistency and imprecision. For secondary outcomes, functional mobility and balance, as assessed by the Timed Up and Go test, showed moderate certainty, whereas stiffness and functional performance were generally rated between low and very low due to similar limitations. These findings indicate that, although statistically significant between-group differences were observed for several outcomes, the overall confidence in the estimated effects remains limited. Consequently, the magnitude and reliability of the observed benefits, particularly those favouring AE, should be interpreted cautiously when considering their clinical relevance. The GRADE assessment therefore underscores the need for further well-designed trials with improved methodological rigour and precision to strengthen the certainty of evidence regarding the comparative effects of AE and LBE in individuals with knee OA.

Future research should prioritise well-powered, multicentre RCTs with robust methodological designs to strengthen the certainty of evidence regarding AE versus LBE in knee OA. Explicit reporting of intervention parameters, including session frequency, duration, intensity, progression strategies and adherence, would facilitate replication and enable meta-analytic synthesis. Standardisation of outcome measures, with clear reporting of scale ranges and application of established MCID thresholds, is crucial to enhance interpretability and clinical relevance. Incorporation of long-term follow-up assessments would provide insight into the sustainability of observed benefits, while subgroup analyses stratified by disease severity (Kellgren–Lawrence grade), sex, age, obesity status, baseline functional capacity, and demographic factors would clarify whether specific patient phenotypes exhibit differential responsiveness to AE versus LBE and would support more personalised prescription. Finally, methodological rigour should be enhanced through assessor blinding, transparent reporting of allocation concealment and consistent use of intention-to-treat analyses, ensuring that future trials yield reliable and generalisable evidence capable of informing clinical practice and optimising exercise prescription for this population. Integration of participant experience, adherence metrics, and standardised reporting of exercise parameters (e.g., frequency, intensity, type, time, volume, and progression), and the collection of socioeconomic and geographic data (e.g., income, education, rurality, proximity to aquatic facilities), will be critical for assessing equity of access and the generalisability of exercise interventions, and for effectively translating RCT findings into real-world practice.

## 5. Conclusions

The evidence included in this update does not establish consistent clinically important superiority of AE over LBE for people with knee OA. Although some unadjusted post-intervention between-group estimates were numerically in favour of AE, most were small, imprecise, inconsistent across studies or instruments, or of uncertain clinical importance, and some may have been influenced by baseline imbalance or differences in intervention content and dose. Confidence in these findings is limited by the predominantly low or very low certainty of evidence, methodological limitations of the included trials, heterogeneity in participant and intervention characteristics, incomplete adherence reporting, and limited longer-term follow-up. The physiological properties of the aquatic environment may improve exercise tolerance for some individuals, particularly those with pain, obesity, or difficulty performing weight-bearing exercise, but this rationale should not be interpreted as evidence that AE is generally more effective than LBE. Exercise modality should therefore be selected according to individual characteristics, preferences, treatment goals, accessibility, resource availability, and clinical context. Further well-designed comparative trials with adequately matched interventions, standardised outcome reporting, established thresholds for clinical importance, and longer-term follow-up are required.

## Figures and Tables

**Figure 1:**
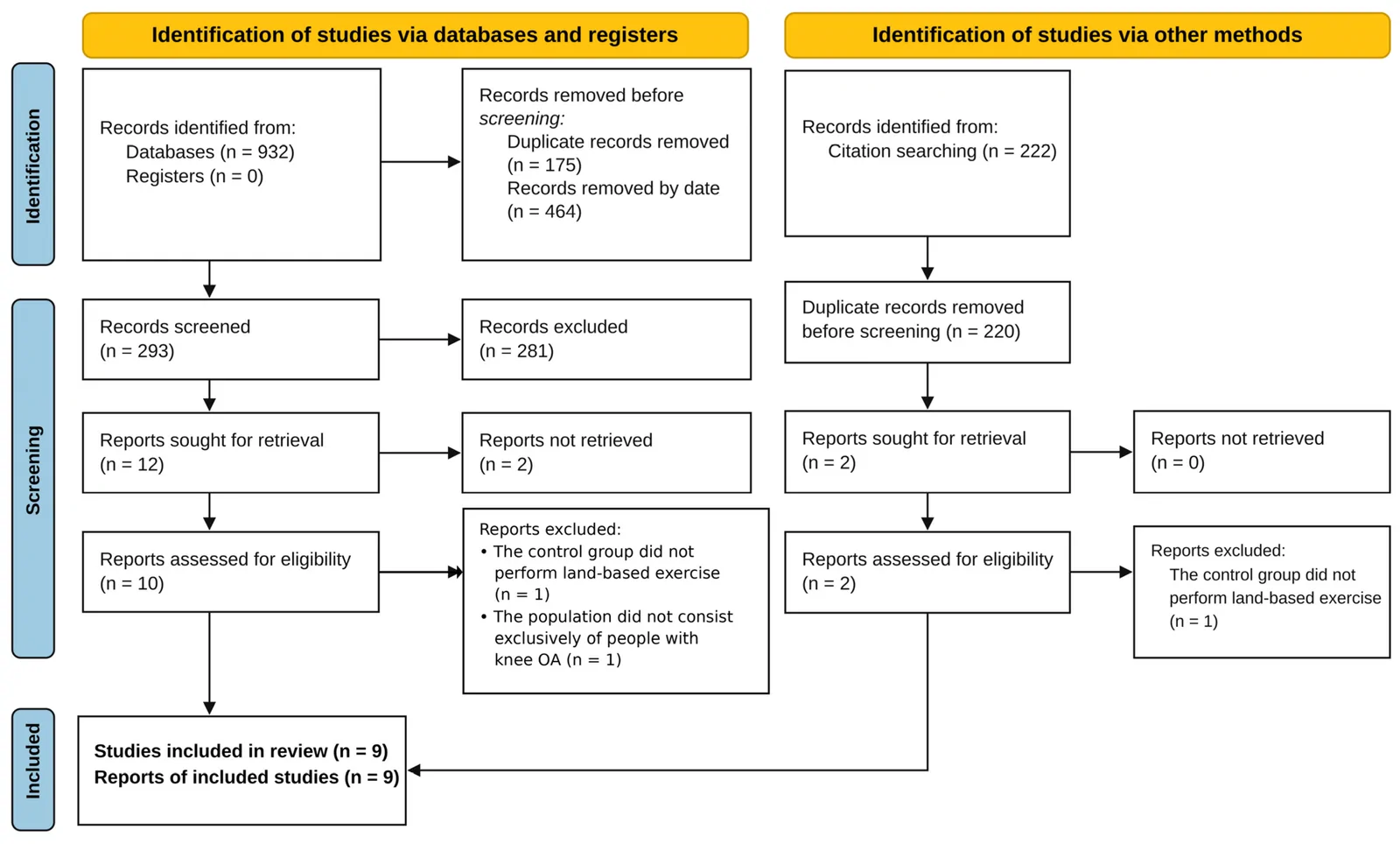
PRISMA 2020 flow diagram illustrating the study selection process through electronic database searching and citation searching.

**Table 1:** Methodological quality assessment of included studies according to the PEDro scale.

Study	#1 ECS	#2 RA	#3 CA	#4 GSB	#5 BS	#6 BT	#7 BA	#8 OD	#9 ITA	#10 RBC	#11 PEVM	Total	Overall Quality
Arrieiro et al. (2019) [88]	Yes	Yes	Yes	Yes	No	No	Yes	Yes	Yes	Yes	Yes	8/10	Good
Assar et al. (2020) [89]	Yes	Yes	Yes	Yes	No	No	Yes	Yes	Yes	Yes	Yes	8/10	Good
Jain & Shinde (2025) [90]	Yes	Yes	Yes	Yes	No	No	Yes	Yes	Yes	Yes	Yes	8/10	Good
Khruakhorn & Chiwarakranon (2021) [91]	Yes	Yes	Yes	Yes	No	No	Yes	Yes	Yes	Yes	Yes	8/10	Good
Krishnan et al. (2022) [92]	Yes	Yes	No	Yes	No	No	No	Yes	No	Yes	Yes	5/10	Fair
Pezeshk et al. (2025) [93]	Yes	Yes	Yes	No	No	No	Yes	Yes	Yes	Yes	Yes	7/10	Good
Shinde & Jain (2025) [94]	Yes	Yes	Yes	Yes	No	No	Yes	Yes	Yes	Yes	Yes	8/10	Good
Slouma et al. (2025) [95]	Yes	Yes	No	No	No	No	Yes	No	No	Yes	Yes	4/10	Fair
Tamin & Loekito (2018) [96]	Yes	Yes	No	Yes	No	No	No	No	No	Yes	Yes	4/10	Fair

ECS—Eligibility Criteria Specified; RA—Random Allocation; CA—Concealed Allocation; GSB—Groups Similar at Baseline; BS—Blinding of Subjects; BT—Blinding of Therapists; BA—Blinding of Assessors; OD—Outcome Data from >85% of Participants; ITA—Intention-to-Treat Analysis; RBC—Reported Between-group Comparisons; PEVM—Point Estimates and Variability Measures. Item 1 (ECS) relates to external validity and is therefore not included in the total PEDro score, which ranges from 0 to 10.

**Table 2:** Characteristics of participants and interventions in the included studies.

Study	N	Intervention *	Female %	Age, Years Mean (SD)	BMI, kg/m^2^ Mean (SD)	Diagnosis	Severity Grade (KL)	Outcomes
Arrieiro et al. (2019) Brazil [88]	AE: 8 LBE: 8	Water-based aerobic walking Land-based aerobic walking	100%	67 (3) 68 (6)	27.56 (2.05) 28.7 (5.39)	Unilateral and bilateral knee OA diagnosed according to the ACR criteria.	I–IV	Pain, physical function, quality of life. Stiffness, functional performance, cardiorespiratory capacity.
Assar et al. (2020) Iran [89]	AE: 12 LBE: 12 CG: 12	Aquatic exercise Total Resistance Exercise Drug regimens	100%	57.5 (6.9) 55.9 (8.6) 63.8 (7.5)	28.5 (3.7) 29.8 (7.2) 23.1 (11.6)	Bilateral knee OA diagnosed according to the ACR criteria.	≥II	Pain. Stiffness, balance, knee instability, range of motion, knee extensors strength.
Jain & Shinde (2025) India [90]	AE: 145 LBE: 145	Aquatic exercise Land-based exercise	89.0% 87.6%	54.32 (11.35) 55.41 (10.272)	26.13 (3.799) 27.02 (3.506)	Bilateral knee OA.	I–II	Pain. Combined symptom and physical function. Functional mobility and balance, lower limb muscle strength, walking speed, joint position sense.
Khruakhorn & Chiwarakranon (2021) Thailand [91]	AE: 17 LBE: 17	Hydrotherapy Land-based exercise	88.24% 94.12%	64.88 (7.44) 57.88 (7.75)	26.34 (2.7) 27.27 (4.38)	Knee OA, diagnosed by an orthopaedic doctor. Unilateral/bilateral not specified.	II–III	Pain, physical function, quality of life. Combined symptom and physical function. Functional mobility and balance, lower limb muscle strength, stiffness, functional performance.
Krishnan et al. (2022) Malaysia [92]	AE: 15 LBE: 15 CG: 15	Aquatic exercise Thera-band (land-based) exercise No intervention	60% 40% 53.3%	51.33 (4.72) 49.47 (5.11) 50.27 (6.34)	33.89 (2.97) 34.41 (2.18) 34.10 (2.97)	Knee OA diagnosed clinically. Unilat-eral/bilateral not specified.	II	Pain. Functional performance.
Pezeshk et al. (2025) Iran [93]	AE: 12 LBE: 12 CG: 12	Water-based exercise Land-based exercise Regular treatment	100%	61.74 (4.6)	30.02 (5.47)	Knee OA diagnosed by orthopaedic doctors and physiotherapists. Unilateral/bilateral not specified.	≥II	Pain. Rectus femoris activity, vastus lateralis activity, biceps femoris activity, semi-membranosus activity, overall muscle activity, extensor/flexor co-contraction, range of motion.
Shinde & Jain (2025) India [94]	AE: 75 LBE: 75	Aquatic exercise Land-based exercise	100%	52.09 (2.472) 50.76 (2.832)	32.10 (1.208) 31.66 (0.9124)	Bilateral knee OA.	I–II	Pain, physical function, quality of life. Combined symptom and physical function. Lower limb muscle strength, hamstring flexibility, hip joint flexibility.
Slouma et al. (2025) Tunisia [95]	AE: 30 LBE: 30	Aquatic exercise Land-based exercise	41.3% 78.6%	50.6 (6.42) 59.1 (10.3)	28.8 (3.8) 31.6 (5.7)	Unilateral and bilateral knee OA diagnosed according to the ACR criteria.	Predominantly ≥ III; 7–13% < III	Pain, physical function, quality of life. Combined symptom and physical function. Stiffness, disease-specific severity, range of motion, quadricep amyotrophy
Tamin & Loekito (2018) Indonesia [96]	AE: 15 LBE: 18	Aquatic exercise Land-based exercise	100% ≥77.78%	59.62 (8.62) 63.4 (7.14)	>25	Knee OA. Uni-lateral/bilateral not specified.	II–III	Quality of life. Cardiorespiratory endurance.

AE: aquatic exercise; LBE: land-based exercise; CG: control group; KL: Kellgren and Lawrence radiographic disease severity scale; ACR: American College of Rheumatology; OA: osteoarthritis; SD: standard deviation. * Intervention labels are reported as designated by the original study authors.

**Table 3:** Description of aquatic and land-based exercise intervention protocols.

Study	Exercise Protocol	Frequency, Intervention Length and Session Duration	Exercise Modality, Session Components, and Setting	Training Load and Progression
Arrieiro et al. (2019) [88]	Water-based aerobic walking	3 sessions/week, 12 weeks, 40–65 min/session	Warm-up (5 min): stretching exercises; Training (30–55 min): walking in a heated pool at target heart rate; Cool-down (5 min): leg movements with buoys and/or holding the pool edge. Pool depth: 1.20 m; heated pool. Supervised by a physiotherapist.	Exercise intensity started at 70–75% HRmax, increased to 75–80% at week 3, progressed to 80–85% at week 5 and maintained until week 12. Training duration started at 30 min and increased by 5 min every 2 weeks, reaching 55 min in the final weeks. Heart rate was monitored every 3 min to maintain the target range.
	Land-based aerobic walking	3 sessions/week, 12 weeks, 40–65 min/session	Warm-up (5 min): stretching exercises; Training (30–55 min): walking on land at target heart rate; Cool-down (5 min): slow walking at a comfortable pace. Supervised by a physiotherapist.	Exercise intensity started at 70–75% HRmax, increased to 75–80% at week 3, progressed to 80–85% at week 5 and maintained until week 12. Training duration started at 30 min and increased by 5 min every 2 weeks, reaching 55 min in the final weeks. Heart rate was monitored every 3 min to maintain the target range.
Assar et al. (2020) [89]	Aquatic exercise	3 sessions/week, 8 weeks, 90 min/session	Warm-up (10 min): walking forward, backward, sideways, and lower-limb stretching; Strength exercises (20 min): hip flexion/extension, hip abduction/adduction, knee flexion/extension, calf raises using water, noodle, elastic band, and sandbag; Aerobic exercises (20 min): stationary running or deep-water running; Step and proprioceptive exercises (20 min): multidirectional gait training and step-up/step-down tasks; Core exercises (10 min); Cool-down (10 min): deep breathing, stretching, and water walking. Water temperature ≈ 32 °C; minimum water depth 1.3 m; sessions supervised by a certified physiotherapist.	Strength, step/proprioceptive, and core exercises: 3 sets of 10 repetitions during weeks 1–4, progressing to 3 sets of 12 repetitions during weeks 5–8, with 10 s rest between sets. Aerobic exercises: 3 sets of 12 repetitions during weeks 1–4, progressing to 3 sets of 15 repetitions during weeks 5–8.
	Total Resistance Exercise	3 sessions/week, 8 weeks, 60 min/session	Warm-up (5–10 min): stretching exercises; TRX training (40–50 min): suspension-based exercises targeting core, hip abductors, and lower-limb muscles; session introduction and technique instruction included; sessions supervised by a TRX specialist with assistant.	TRX exercises performed at 3 sets of 10 repetitions during weeks 1–2, progressing to 3 sets of 15 repetitions during weeks 3–4; similar repetition progression applied during weeks 5–8, with increasing exercise difficulty achieved by narrowing base of support, altering body angle relative to gravity, and adding instability rather than external load increments.
Jain & Shinde (2025) [90]	Aquatic exercise	3 sessions/week, 8 weeks, 40–50 min/session	Warm-up (10 min): aquatic running with belt; walking forward, backward, and sideways with kickboard. Strength training (10 min): hip flexion–extension, hip hyperextension, hip abduction–adduction, knee flexion–extension, kickback performed against water resistance, with aquatic cuffs introduced from week 4. Balance and proprioception training: marching on the spot; knee-to-chest; cross-country skiing; tip-toe walking; semi-squats and lunges; step-up/down/forward/backward; multidirectional walking drills; figure-of-eight walking; single- and double-leg stance tasks using noodles, kickboards, aqua dumbbells, and ball throwing. Cool-down (10 min): supported cycling against wall and stretching. Pool temperature 36 °C; water depth at median sternum; sessions supervised by a licensed physiotherapist.	Progressive increase in repetitions, duration, sets, and task difficulty from weeks 1 to 8, including progression of walking distance, repetitions per minute, hold times, introduction of aquatic cuffs from week 4, increased sets, longer balance holds, and progression to eyes-closed balance tasks.
	Land-based exercise	3 sessions/week, 8 weeks, 40–50 min/session	Warm-up (10 min): cycling on stationary bike; walking forward, backward, and sideways. Strength training (10 min): hip flexion–extension, hip hyperextension, hip abduction–adduction, knee flexion–extension (sitting and standing), kickback exercises using therabands of low to medium resistance. Balance and proprioception training: marching on the spot; knee-to-chest; cross-country skiing; tip-toe walking; semi-squats and lunges; step-up/down/forward/backward; multidirectional walking drills (progressing to eyes closed from week 4); figure-of-eight walking with and without wand; single- and double-leg stance tasks on hard surfaces; ball-throwing tasks. Cool-down (10 min): floor-based relaxation and stretching. Sessions supervised by a licensed physiotherapist.	Progressive increase in repetitions, steps, sets, resistance, and balance task difficulty from weeks 1 to 8, including increased steps and repetitions, increased sets, progression of theraband resistance, longer hold times, and progression to eyes-closed balance exercises from week 4.
Khruakhorn & Chiwarakranon (2021) [91]	Hydrotherapy	3 sessions/week, 6 weeks, 45–60 min/session	Stretching exercises for lower-limb muscles (hamstrings, quadriceps, calf, hip abductors/adductors): hold 10 s, 10 repetitions. Progressive strengthening exercises for lower limb and trunk using water resistance and flotation devices (noodles): 3 sets of 15 repetitions. Functional movements including single-leg swings (forward, backward, diagonal), hip and knee flexion–extension, knee extension against water resistance, semi-squats, jogging in water for calf strengthening, and deep-water cycling with flotation noodles (10 min in phase 2; 15 min in phase 3). Supervised sessions in a hydrotherapy pool, water temperature 32–33 °C, depth not reported.	Programme divided into phases; phase changed every six exercise sessions. Progression involved increasing number of exercises, adding resistance via water properties and flotation devices, increasing movement speed as tolerated, and increasing cycling duration from 10 to 15 min in later phases.
	Land-based exercise	3 sessions/week, 6 weeks, 45–60 min/session	Stretching exercises for lower-limb muscles (hamstrings, quadriceps, calf, hip abductors/adductors): hold 10 s, 10 repetitions. Progressive strengthening exercises for lower limb and trunk performed on an exercise mat using body weight and elastic bands: 3 sets of 15 repetitions. Functional movements including single-leg swings (forward, backward, diagonal), hip and knee flexion–extension, knee extension with elastic resistance, semi-squats, tiptoe exercises for calf strengthening, and stationary cycling on land (10 min in phase 2; 15 min in phase 3). All sessions were supervised by a physiotherapist.	Programme divided into phases; phase changed every six exercise sessions. Progression involved increasing the number of exercises, adding resistance using elastic bands, increasing movement speed as tolerated, and increasing cycling duration from 10 to 15 min in later phases.
Krishnan et al. (2022) [92]	Aquatic exercise	2 sessions/week, 8 weeks, 60 min/session	Warm-up (10 min); aquatic exercise (40 min); cool-down, relaxation exercises, self-care, and free water activity (10 min). Aquatic exercises included forward and backward walking, knee flexion and extension (standing), side lunges, jogging, side stepping, standing knee lift, hip kickers, leg balance (right and left), deep-water cycling using a woggle, pool plank, and wall push-ups. Sessions were supervised by an aquatic exercise therapist in a hydrotherapy pool with water depth adjusted to mid-sternum level (~1.2 m) and water temperature maintained at 36 °C.	Exercise intensity and frequency progressively increased weekly. Intensity was monitored using the Borg Rating of Perceived Exertion, targeting 13–17 (approximately 60–70% of maximum heart rate). Progression was achieved by increasing repetitions, sets, distance, duration, and exercise complexity as detailed in the weekly programme.
	Thera-band (land-based) exercise	2 sessions/week, 8 weeks, 60 min/session	Warm-up (10 min); Thera-band resistance exercise programme (40 min); cool-down (10 min). Exercises included hip flexion and extension (standing), hip abduction and adduction (standing), quick kicks, terminal knee extension, knee flexion and extension (sitting and prone), mini squat, leg press, squat, lunge, upper body mobilisation (lateral raise, front raise, overhead press), and shoulder diagonal movements. Sessions were supervised.	Progressive overload was applied across the 8 weeks by increasing repetitions and sets weekly. Resistance level was self-selected using colour-coded Thera-bands (yellow, red, green, or blue). Training intensity was monitored using the Borg Rating of Perceived Exertion, with progression based on tolerance and repetition maximum.
Pezeshk et al. (2025) [93]	Water-based exercise	3 sessions/week, 8 weeks, 60 min/session	Warm-up (10 min): slow walking and quadriceps, hamstring, and gastrocnemius stretching. Main exercises (45 min): stretching; isometric resistance exercises (ankle dorsiflexion and plantar flexion held for 6 s); isotonic strengthening exercises for hip, knee, and trunk (e.g., hip abduction/adduction, knee flexion/extension, heel raises, cycling movements); gait training (forward, backward, sideways walking, and walking with knee lift). Cool-down (5 min): lower-limb stretching. The intervention was administered by qualified physiotherapists and exercise trainers. Sessions were conducted in a therapeutic pool at 32 °C, with water depth at anterior superior iliac spine.	Fixed programme across 18 sessions. Exercise volume defined by prescribed repetitions and holding times (isometric contractions held for 6 s; stretching held for 20 s with 7–10 repetitions; isotonic exercises performed for 20–40 repetitions). No explicit progressive overload or systematic increase in intensity, repetitions, or resistance reported.
	Land-based exercise	3 sessions/week, 8 weeks, 60 min/session	Warm-up (10 min): slow walking and quadriceps, hamstring, and gastrocnemius stretching. Main exercises (45 min): stretching; isometric resistance exercises (ankle dorsiflexion and plantar flexion held for 6 s); isotonic strengthening exercises for hip, knee, and trunk (e.g., bridges, knee extension/flexion, hip abduction/adduction, heel raises, cycling on stationary bicycle); gait training (forward, backward, sideways walking, and walking with knee lift). Cool-down (5 min): lower-limb stretching. The intervention was administered by qualified physiotherapists and exercise trainers. Sessions were conducted in a gymnasium.	Fixed programme across 18 sessions. Exercise volume defined by prescribed repetitions and holding times (isometric contractions held for 6 s; stretching held for 20 s with 7–10 repetitions; isotonic exercises performed for 20–40 repetitions). No explicit progressive overload or systematic increase in intensity, repetitions, or resistance reported.
Shinde & Jain (2025) [94]	Aquatic exercise	3 sessions/week, 8 weeks, 55–60 min/session	Warm-up (5 min); Main aquatic exercises (45–50 min): flexibility exercises, upper and lower body training, aerobic/endurance training, strength training, balance and proprioception training; Cool-down (5 min); conducted in warm water (36 °C) at 1.2 m depth; supervised by trained physiotherapists.	Intensity and frequency adjusted to participants’ abilities (self-selected walking pace and repetition rate); progressively increased over first two weeks; conditioning intensity 13–17 Borg scale (60–70% HRmax).
	Land-based exercise	3 sessions/week, 8 weeks, 40–50 min/session	Warm-up (5 min); Land-based exercises (40 min): flexibility, upper and lower body strengthening, aerobic/endurance, balance and proprioception training; Cool-down (5 min); conducted in a gym under physiotherapist supervision.	Exercise intensity and frequency adjusted according to participants’ abilities; progressively increased over first two weeks; sets, repetitions, and distances similar in structure to aquatic exercises but performed on land.
Slouma et al. (2025) [95]	Aquatic exercise	3 sessions/week, 8 weeks, 60 min/session	Warm-up (15 min); Exercises to gain joint mobility; Strengthening and endurance exercises; Proprioceptive training; Water temperature 26 °C; Immersion to xiphoid level; Supervised by sports educators.	Not reported.
	Land-based exercise	3 sessions/week, 8 weeks, 50 min/session	Passive then assisted active mobilisation; Static strengthening of quadriceps and hamstrings; Hamstring stretching; Proprioceptive training on stable and unstable surfaces; Sessions were supervised.	Not reported.
Tamin & Loekito (2018) [96]	Aquatic exercise	3 sessions/week, 8 weeks, 50 min/session	Aerobic exercise with quick movements in four directions (forward, backward, left, right); Strengthening exercises using ankle weights; Water temperature 32 °C; Depth approximately at the xiphoid process; Sessions supervised by a certified physiotherapist.	Aerobic exercise: Week 1—1 repetition; Week 2—2 repetitions; Weeks 3–4—3 repetitions; Weeks 5–6—4 repetitions; Weeks 7–8—5 repetitions. Strengthening exercise: Weeks 1–4—3 sets for each muscle, 6 repetitions, hold 6 s; Weeks 5–8—3 sets for each muscle, 16 repetitions, hold 6 s.
	Land-based exercise	3 sessions/week, 8 weeks, 50 min/session	Aerobic exercise using a static bicycle at 50 RPM; Strengthening exercises targeting lower limb muscles; Sessions supervised by a certified physiotherapist.	Aerobic exercise: Week 1—15 min at 0 KP; Week 2—30 min at 0 KP; Weeks 3–4—45 min at 0 KP; Week 5—60 min at 0 KP; Week 6—15 min at 1 KP; Week 7—30 min at 1 KP; Week 8—45 min at 1 KP. Strengthening exercise: 3 sets for each muscle, 5 repetitions, hold 6 s at 50% of maximum load, progressed according to exercise test results.

HRmax: maximum heart rate; TRX: Total Resistance Exercise; RPM: revolutions per minute. Intervention comparability based on exercise content and dose: closely matched, [88,90,91,93]; partially matched, [94,95]; fundamentally different, [89,92,96]. This classification was conducted descriptively from the intervention characteristics reported by the original studies.

**Table 4:** Primary outcomes by intervention group, presenting pre- and post-intervention values, unadjusted post-intervention between-group mean differences, and the overall certainty of the evidence (GRADE).

Outcome	Instrument	Units/Scale	Study	Group	N	Pre-Intervention (T0) Mean (SD)	Post-Intervention (T1) Mean (SD)	Unadjusted Post-Intervention Between-Group Mean Difference (95% CI) *	GRADE Overall Certainty of the Evidence ‡‡
Pain	VAS rest	Not reported (lower is better)	Jain & Shinde (2025) [90]	AE—left knee	145	3.67 (1.29)	1.76 (0.58)	−0.60 [−0.79, −0.41] **	⨁⨁◯◯ Low
				LBE—left knee	145	3.90 (1.12)	2.36 (1.04)		
				AE—right knee	145	3.78 (1.02)	1.55 (0.53)	−0.59 [−0.77, −0.41] **	
				LBE—right knee	145	3.51 (1.17)	2.14 (0.97)		
		Not reported (lower is better)	Shinde & Jain (2025) [94]	AE	75	4.82 (0.76)	2.16 (0.65)	−0.52 [−0.72, −0.32] **	
				LBE	75	4.56 (0.8094)	2.68 (0.5964)		
		Centimetres: 0 (best) to 10 (worst)	Slouma et al. (2025) [95]	AE	30	1.8 (2.5)	0.5 (0.9)	−0.77 [−1.44, −0.10] **	
				LBE	30	2.47 (2.3)	1.27 (1.6)		
	VAS activity	Centimetres: 0 (best) to 10 (worst)	Assar et al. (2020) [89]	AE	12	7.1 (2.7)	3.1 (1.6)	−0.30 [−1.94, 1.34]	⨁◯◯◯ Very low
				LBE	12	7.6 (2.4)	3.4 (2.2)		
		Not reported (lower is better)	Jain & Shinde (2025) [90]	AE—left knee	145	6.22 (0.48)	2.27 (1.00)	−0.44 [−0.69, −0.19] **	
				LBE—left knee	145	6.31 (0.59)	2.71 (1.19)		
				AE—right knee	145	6.16 (0.55)	2.19 (0.92)	−0.32 [−0.54, −0.10] **	
				LBE—right knee	145	6.07 (0.51)	2.51 (0.97)		
		Not reported (lower is better)	Shinde & Jain (2025) [94]	AE	75	6.26 (0.60)	3.04 (0.72)	−0.50 [−0.71, −0.29] **	
				LBE	75	6.90 (0.8411)	3.54 (0.5994)		
		Centimetres: 0 (best) to 10 (worst)	Slouma et al. (2025) [95]	AE	30	7.6 (1.8)	4.8 (1.7)	−1.80 [−2.82, −0.78] **	
				LBE	30	7.7 (2.0)	6.6 (2.2)		
	VAS unspecified	Points: 0 (best) to 10 (worst)	Krishnan et al. (2022) [92]	AE	15	5.60 (0.91)	2.87 (0.52)	−0.13 [−0.62, 0.36]	⨁⨁⨁◯ Moderate
				LBE	15	5.40 (0.63)	3.00 (0.76)		
	WOMAC pain	Points: range not reported (lower is better)	Arrieiro et al. (2019) [88]	AE	8	152.25 (92.50) §	71.58 (108.94) §	−27.98 [−154.17, 98.21]	⨁◯◯◯ Very low
				LBE	8	171.15 (106.89) §	99.56 (125.39) §		
		Points: range not reported (lower is better)	Khruakhorn & Chiwarakranon (2021) [91]	AE	17	17.53 (12.44)	7.47 (6.85)	−0.47 [−6.16, 5.22]	
				LBE	17	18.82 (10.89)	7.94 (9.22)		
		Points: range not reported (lower is better)	Slouma et al. (2025) [95]	AE	30	11.3 (3.2)	5.4 (2.4)	−6.30 [−8.18, −4.42] **	
				LBE	30	14.2 (5.1)	11.7 (4.5)		
	KOOS pain	Points: 0 (worst) to 100 (best)	Pezeshk et al. (2025) [93]	AE	12	32.3 (22.2)	57.02 (24)	2.82 [−15.88, 21.52]	⨁◯◯◯ Very low
				LBE	12	37.8 (24.6)	54.2 (19.9)		
Physical function	WOMAC physical function	Points: range not reported (lower is better)	Arrieiro et al. (2019) [88]	AE	8	622.49 (383.34) §	245.88 (312.10) §	−53.97 [−392.34, 284.40]	⨁◯◯◯ Very low
				LBE	8	530.13 (325.67) §	299.85 (318.89) §		
		Points: range not reported (lower is better)	Khruakhorn & Chiwarakranon (2021) [91]	AE	17	51.47 (36.43)	20.24 (18.81)	−4.11 [−21.13, 12.91]	
				LBE	17	64.29 (32.41)	24.35 (28.61)		
		Points: range not reported (lower is better)	Slouma et al. (2025) [95]	AE	30	34.0 (11.2)	17.6 (8.1)	−22.70 [−28.64, −16.76] **	
				LBE	30	45.2 (15.7)	40.3 (14.0)		
Combined symptom and physical function	WOMAC total	Points: range not reported (lower is better)	Jain & Shinde (2025) [90]	AE	145	60.37 (7.22)	49.42 (6.52)	−4.37 [−5.99, −2.75] **	⨁◯◯◯ Very low
				LBE	145	58.50 (8.40)	53.79 (7.45)		
		Points: 0 (best) to 220 (worst)	Khruakhorn & Chiwarakranon (2021) [91]	AE	17	75.06 (50.71)	31.65 (27.56)	−4.11 [−28.72, 20.50]	
				LBE	17	90.71 (44.33)	35.76 (41.15)		
		Points: range not reported (lower is better)	Shinde & Jain (2025) [94]	AE	75	68.64 (1.035)	50.2 (0.8054)	−11.42 [−11.70, −11.14] **	
				LBE	75	66.01 (0.762)	61.62 (0.8970)		
		Points: range not reported (lower is better)	Slouma et al. (2025) [95]	AE	30	49.6 (15.8)	25.1 (11.1)	−31.40 [−39.74, −23.06] **	
				LBE	30	64.9 (22.7)	56.5 (19.8)		
Quality of life	WHOQOL-BREF physical	Points: 7 (worst) to 35 (best)	Khruakhorn & Chiwarakranon (2021) [91]	AE	17	23.06 (3.25)	27 (2.83)	−0.24 [−2.45, 1.97]	⨁⨁◯◯ Low
				LBE	17	23.76 (3.93)	27.24 (3.46)		
	WHOQOL-BREF mental	Points: 6 (worst) to 30 (best)	Khruakhorn & Chiwarakranon (2021) [91]	AE	17	22 (4.61)	24.29 (1.69)	0.11 [−2.24, 2.46]	⨁⨁◯◯ Low
				LBE	17	23.24 (4.09)	24.18 (4.33)		
	WHOQOL-BREF social	Points: 3 (worst) to 15 (best)	Khruakhorn & Chiwarakranon (2021) [91]	AE	17	10.29 (3.22)	10.71 (1.11)	−0.58 [−1.80, 0.64]	⨁⨁◯◯ Low
				LBE	17	10.12 (1.97)	11.29 (2.17)		
	WHOQOL-BREF environments	Points: 8 (worst) to 40 (best)	Khruakhorn & Chiwarakranon (2021) [91]	AE	17	28.53 (3.28)	31.06 (2.79)	0.88 [−1.88, 3.64]	⨁⨁◯◯ Low
				LBE	17	29.65 (4.99)	30.18 (4.77)		
	WHOQOL-BREF total	Points: 26 (worst) to 130 (best)	Khruakhorn & Chiwarakranon (2021) [91]	AE	17	83.88 (10.99)	93.06 (5.80)	0.18 [−6.61, 6.97]	⨁⨁◯◯ Low
				LBE	17	86.76 (11.91)	92.88 (12.23)		
	SF-12 physical domain	Points: range not reported (higher is better)	Slouma et al. (2025) [95]	AE	30	38.2 (7.8)	43.6 (4.9)	10.70 [7.48, 13.92] **	⨁⨁⨁⨁ High
				LBE	30	30.6 (8.4)	32.9 (7.3)		
	SF-12 mental domain	Points: range not reported (higher is better)	Slouma et al. (2025) [95]	AE	30	35.9 (9.6)	46.9 (9.0)	5.80 [0.69, 10.91] **	⨁⨁⨁◯ Moderate
				LBE	30	34.8 (10.1)	41.1 (10.7)		
	SF-36 physical functioning	Points: 0 (worst) to 100 (best)	Arrieiro et al. (2019) [88]	AE	8	41.88 (21.52) ‡	70.00 (9.98) ‡	−5.00 [−15.70, 5.70]	⨁◯◯◯ Very low
				LBE	8	54.38 (14.48) ‡	75.00 (9.98) ‡		
		Points: 0 (worst) to 100 (best)	Shinde & Jain (2025) [94]	AE	75	53.8 (0.56)	67.32 (0.46)	4.86 [4.71, 5.02] **	
				LBE	75	56.62 (0.65)	62.46 (0.50)		
		Points: 0 (worst) to 100 (best)	Tamin & Loekito (2018) [96]	AE	15	50.00 (20.12) †	49.14 (18.68) †	−21.94 [−35.90, −7.98] **	
				LBE	18	59.22 (23.33) †	71.08 (20.58) †		
	SF-36 role-physical	Points: 0 (worst) to 100 (best)	Arrieiro et al. (2019) [88]	AE	8	46.88 (36.44) ‡	81.25 (11.57) ‡	−9.38 [−22.55, 3.79]	⨁◯◯◯ Very low
				LBE	8	50.00 (29.86) ‡	90.63 (12.92) ‡		
		Points: 0 (worst) to 100 (best)	Shinde & Jain (2025) [94]	AE	75	62.44 (0.72)	73.29 (0.69)	5.27 [4.99, 5.55] **	
				LBE	75	60.8 (1.16)	68.02 (1.02)		
		Points: 0 (worst) to 100 (best)	Tamin & Loekito (2018) [96]	AE	15	50.00 (28.75) †	58.20 (28.75) †	−26.10 [−46.23, −5.97] **	
				LBE	18	58.58 (27.45) †	84.30 (27.45) †		
	SF-36 bodily pain	Points: 0 (worst) to 100 (best)	Arrieiro et al. (2019) [88]	AE	8	41.88 (25.86) ‡	65.00 (9.67) ‡	1.62 [−7.54, 10.78]	⨁◯◯◯ Very low
				LBE	8	37.13 (18.10) ‡	63.38 (7.07) ‡		
		Points: 0 (worst) to 100 (best)	Shinde & Jain (2025) [94]	AE	75	52.98 (0.64)	67.76 (0.43)	4.78 [4.60, 4.96] **	
				LBE	75	55.65 (0.72)	62.98 (0.64)		
		Points: 0 (worst) to 100 (best)	Tamin & Loekito (2018) [96]	AE	15	56.64 (12.94) †	60.51 (19.40) †	−4.64 [−19.33, 10.05]	
				LBE	18	55.54 (10.29) †	65.15 (21.96) †		
	SF-36 general health	Points: 0 (worst) to 100 (best)	Arrieiro et al. (2019) [88]	AE	8	51.63 (11.09) ‡	68.25 (10.91) ‡	5.00 [−5.84, 15.84]	⨁◯◯◯ Very low
				LBE	8	44.63 (13.23) ‡	63.25 (9.17) ‡		
		Points: 0 (worst) to 100 (best)	Shinde & Jain (2025) [94]	AE	75	69.77 (0.78)	78.6 (0.47)	4.34 [4.13, 4.55] **	
				LBE	75	68.48 (0.77)	74.26 (0.77)		
		Points: 0 (worst) to 100 (best)	Tamin & Loekito (2018) [96]	AE	15	55.70 (15.81) †	62.42 (10.06) †	−3.66 [−12.66, 5.34]	
				LBE	18	61.42 (15.10) †	66.08 (15.10) †		
	SF-36 vitality	Points: 0 (worst) to 100 (best)	Arrieiro et al. (2019) [88]	AE	8	49.50 (11.60) ‡	74.38 (13.75) ‡	0.00 [−13.34, 13.34]	⨁◯◯◯ Very low
				LBE	8	57.50 (14.88) ‡	74.38 (10.83) ‡		
		Points: 0 (worst) to 100 (best)	Shinde & Jain (2025) [94]	AE	75	70.46 (1.03)	80.89 (0.55)	2.76 [2.54, 2.98] **	
				LBE	75	71.26 (0.68)	78.13 (0.79)		
		Points: 0 (worst) to 100 (best)	Tamin & Loekito (2018) [96]	AE	15	65.00 (8.62) †	73.28 (11.50) †	−0.01 [−8.96, 8.94]	
				LBE	18	64.84 (13.99) †	73.29 (13.72) †		
	SF-36 social functioning	Points: 0 (worst) to 100 (best)	Arrieiro et al. (2019) [88]	AE	8	56.25 (35.32) ‡	65.63 (16.01) ‡	−17.18 [−35.24, 0.88]	⨁◯◯◯ Very low
				LBE	8	57.81 (18.81) ‡	82.81 (17.60) ‡		
		Points: 0 (worst) to 100 (best)	Shinde & Jain (2025) [94]	AE	75	78.82 (0.94)	88.37 (0.48)	3.49 [3.28, 3.70] **	
				LBE	75	80.08 (0.86)	84.88 (0.77)		
		Points: 0 (worst) to 100 (best)	Tamin & Loekito (2018) [96]	AE	15	85.35 (10.78) †	75.00 (14.37) †	−17.15 [−27.21, −7.09] **	
				LBE	18	81.30 (17.70) †	92.15 (13.72) †		
	SF-36 role-emotional	Points: 0 (worst) to 100 (best)	Arrieiro et al. (2019) [88]	AE	8	58.33 (46.26) ‡	83.33 (35.60) ‡	−8.32 [−39.09, 22.45]	⨁◯◯◯ Very low
				LBE	8	49.99 (35.60) ‡	91.65 (15.44) ‡		
		Points: 0 (worst) to 100 (best)	Shinde & Jain (2025) [94]	AE	75	77.7 (0.80)	83.16 (0.77)	3.42 [3.17, 3.67] **	
				LBE	75	74.64 (0.69)	79.74 (0.77)		
		Points: 0 (worst) to 100 (best)	Tamin & Loekito (2018) [96]	AE	15	82.79 (28.75) †	82.79 (28.75) †	−6.74 [−24.51, 11.03]	
				LBE	18	84.30 (27.45) †	89.53 (18.31) †		
	SF-36 mental health	Points: 0 (worst) to 100 (best)	Arrieiro et al. (2019) [88]	AE	8	67.50 (18.55) ‡	77.50 (17.63) ‡	−4.50 [−19.46, 10.46]	⨁◯◯◯ Very low
				LBE	8	63.00 (16.66) ‡	82.00 (5.66) ‡		
		Points: 0 (worst) to 100 (best)	Shinde & Jain (2025) [94]	AE	75	75.85 (0.71)	81.8 (0.40)	5.88 [5.71, 6.05] **	
				LBE	75	76.18 (0.72)	75.92 (0.65)		
		Points: 0 (worst) to 100 (best)	Tamin & Loekito (2018) [96]	AE	15	70.07 (12.65) †	87.31 (8.05) †	2.57 [−3.80, 8.94]	
				LBE	18	80.74 (12.08) †	84.74 (9.88) †		

AE: aquatic exercise; LBE: land-based exercise; CI: confidence interval; SD: standard deviation; VAS: Visual Analogue Scale; WOMAC: Western Ontario and McMaster Universities Osteoarthritis Index; KOOS: Knee Injury and Osteoarthritis Outcome Score; SF-12: Short Form 12 Health Survey; SF-36: Short Form 36 Health Survey; WHOQOL-BREF: World Health Organization Quality of Life—Bref (abbreviated version); NR: not reported; NA: not assessed. * Unadjusted post-intervention mean differences and 95% confidence intervals were calculated by the review authors from the reported group means, standard deviations, and sample sizes. These estimates do not account for baseline differences in the outcome or other participant characteristics and should be interpreted as descriptive between-group comparisons rather than baseline-adjusted treatment effects. † Mean and standard deviation were estimated from sample size, median, minimum and maximum. ‡ Standard deviations were estimated using the formula SD = SE × √n. § Data were extracted from published graphs. ** Indicate statistically significant between-group differences (95% CI does not include zero). ‡‡ The overall certainty of evidence was evaluated across the five GRADE domains (risk of bias, inconsistency, indirectness, imprecision, and publication bias) and classified as high (⨁⨁⨁⨁), moderate (⨁⨁⨁◯), low (⨁⨁◯◯), or very low (⨁◯◯◯). Between-group mean differences were always calculated as AE minus LBE. For outcomes where lower scores indicate improvement, negative values favour aquatic exercise and positive values favour land-based exercise. For outcomes where higher scores indicate improvement, positive values favour aquatic exercise and negative values favour land-based exercise.

**Table 5:** Secondary outcomes by intervention group, presenting pre- and post-intervention values, unadjusted post-intervention between-group mean differences, and the overall certainty of the evidence (GRADE).

Outcome	Instrument	Units/Scale	Study	Group	N	Pre-Intervention (T0) Mean (SD)	Post-Intervention (T1) Mean (SD)	Unadjusted Post-Intervention Between-Group Mean Difference (95% CI) *	GRADE Overall Certainty of the Evidence ‡‡
Stiffness	WOMAC stiffness	Points: range not reported (lower is better)	Arrieiro et al. (2019) Brazil [88]	AE LBE	8 8	68.79 (50.84) § 62.43 (78.24) §	25.12 (43.24) § 31.36 (45.47) §	−6.24 [−53.83, 41.35]	⨁◯◯◯ Very low
		Points: 0 (best) to 8 (worst)	Assar et al. (2020) Iran [89]	AE LBE	12 12	4.9 (2.1) 4.4 (2.3)	2.9 (0.02) 2.8 (2.1)	0.10 [−1.23, 1.43]	
		Points: range not reported (lower is better)	Khruakhorn & Chiwarakranon (2021) Thailand [91]	AE LBE	17 17	6.06 (4.91) 7.59 (6.12)	3.94 (4.66) 3.47 (4.53)	0.47 [−2.74, 3.68]	
		Points: range not reported (lower is better)	Slouma et al. (2025) Tunisia [95]	AE LBE	30 30	4.3 (2.6) 5.5 (2.5)	2.0 (1.5) 4.5 (2.3)	−2.50 [−3.51, −1.49] **	
Functional performance	6 min walk test	Metres (higher is better)	Arrieiro et al. (2019) Brazil [88]	AE LBE	8 8	457.44 (58.70) § 451.13 (40.83) §	507.97 (58.70) § 521.50 (56.14) §	−13.53 [−75.13, 48.07]	⨁◯◯◯ Very low
		Metres (higher is better)	Krishnan et al. (2022) Malaysia [92]	AE LBE	15 15	305.87 (32.66) 295.57 (28.48)	354.80 (32.68) 321.40 (32.25)	33.40 [9.12, 57.68] **	
	Stair test (4 steps)	Seconds (lower is better)	Arrieiro et al. (2019) Brazil [88]	AE LBE	8 8	6.76 (1.97) § 7.45 (1.01) §	6.24 (1.47) § 6.36 (0.88) §	−0.12 [−1.45, 1.21]	⨁⨁◯◯ Low
		Seconds (lower is better)	Khruakhorn & Chiwarakranon (2021) Thailand [91]	AE LBE	17 17	10.68 (5.85) 8.37 (4.49)	6.86 (3.41) 8.10 (7.51)	−1.24 [−5.39, 2.91]	
Cardiorespiratory capacity	Maximum oxygen consumption	mL·kg^−1^·min^−1^ (higher is better)	Arrieiro et al. (2019) Brazil [88]	AE LBE	8 8	16.35 (3.85) § 16.79 (2.50) §	17.97 (5.10) § 18.15 (2.19) §	−0.18 [−4.58, 4.22]	⨁⨁◯◯ Low
	Anaerobic threshold	mL·kg^−1^·min^−1^ (higher is better)	Arrieiro et al. (2019) Brazil [88]	AE LBE	8 8	31.59 (6.54) § 29.87 (6.54) §	35.44 (4.36) § 31.94 (4.53) §	3.50 [−1.27, 8.27]	⨁⨁◯◯ Low
Cardiorespiratory endurance	Borg scale	Points: 6 (no exertion) to 20 (maximal exertion)	Tamin & Loekito (2018) Indonesia [96]	AE LBE	15 18	NR NR	NR NR	NR	NA
	Borg category ratio 10 scale	Points: 0 (no exertion) to 10 (maximal exertion)	Tamin & Loekito (2018) Indonesia [96]	AE LBE	15 18	NR NR	NR NR	NR	NA
Balance	Berg Balance Scale	Points: 0 (worst) to 56 (best)	Assar et al. (2020) Iran [89]	AE LBE	12 12	41.3 (8.3) 37.6 (8.9)	52.5 (4.88) 47.9 (7.88)	4.60 [−1.01, 10.21]	⨁⨁◯◯ Low
Knee instability	Felson’s questionnaire	Points: 0 (worst) to 5 (best)	Assar et al. (2020) Iran [89]	AE LBE	12 12	1.6 (1.4) 2.1 (1.6)	3.9 (1.2) 4.7 (0.6)	−0.80 [−1.62, 0.02]	⨁⨁⨁◯ Moderate
Range of motion	Bubble inclinometer	Degrees (higher is better)	Assar et al. (2020) Iran [89]	AE LBE	12 12	110.4 (6.7) 109.7 (5.5)	121.9 (8.5) 129.4 (4.9)	−7.50 [−13.46, −1.54] **	⨁⨁◯◯ Low
	2D video-based kinematics	Degrees (higher is better)	Pezeshk et al. (2025) Iran [93]	AE LBE	12 12	18.4 (6.05) 18.4 (6.05)	25.44 (5.7) 25.58 (6.7)	−0.14 [−5.41, 5.13]	⨁⨁⨁◯ Moderate
	Goniometer	Degrees (higher is better)	Slouma et al. (2025) Tunisia [95]	AE LBE	30 30	133.3 (8.8) 123.1 (13.7)	135.4 (7.7) 124.4 (13.0)	11.0 [5.45, 16.55] **	⨁⨁⨁◯ Moderate
Lower limb muscle strength	Manual muscle testing	Points: 0 (worst) to 5 (best)	Shinde & Jain (2025) India [94]	AE—Right HF LBE—Right HF	75 75	3.10 (0.48) 3.04 (0.4172)	4.08 (0.6098) 3.26 (0.4452)	0.82 [0.65, 0.99] **	⨁⨁⨁⨁ High
				AE—Left HF LBE—Left HF	75 75	3.05 (0.4619) 3.17 (0.3811)	4.02 (0.6773) 3.4 (0.5199)	0.62 [0.43, 0.81] **	⨁⨁⨁⨁ High
				AE—Right HE LBE—Right HE	75 75	3.18 (0.45) 3.02 (0.56)	4.04 (0.5560) 3.34 (0.50)	0.70 [0.53, 0.87] **	⨁⨁⨁⨁ High
				AE—Left HE LBE—Left HE	75 75	3.09 (0.4979) 3.05 (0.5670)	4.06 (0.5022) 3.8 (0.5452)	0.26 [0.09, 0.43] **	⨁⨁⨁⨁ High
				AE—Right KF LBE—Right KF	75 75	3.16 (0.4943) 3.10 (0.5087)	4.2 (0.5452) 3.70 (0.5139)	0.50 [0.33, 0.67] **	⨁⨁⨁⨁ High
				AE—Left KF LBE—Left KF	75 75	3.09 (0.4403) 3.02 (0.4925)	4.10 (0.6057) 3.29 (0.4584)	0.81 [0.64, 0.98] **	⨁⨁⨁⨁ High
				AE—Right KE LBE—Right KE	75 75	3.06 (0.5022) 3.0 (0.4650)	4.17 (0.5544) 3.88 (0.6568)	0.29 [0.09, 0.49] **	⨁⨁⨁⨁ High
				AE—Left KE LBE—Left KE	75 75	3.10 (0.3881) 3.04 (0.5051)	4.13 (0.4746) 3.86 (0.6003)	0.27 [0.10, 0.44] **	⨁⨁⨁⨁ High
				AE—Right PF LBE—Right PF	75 75	3.04 (0.4484) 3.01 (0.4189)	4.2 (0.5927) 3.24 (0.4603)	0.96 [0.79, 1.13] **	⨁⨁⨁⨁ High
				AE—Left PF LBE—Left PF	75 75	3.04 (0.4316) 3.02 (0.4341)	4.09 (0.5244) 3.72 (0.6485)	0.37 [0.18, 0.56] **	⨁⨁⨁⨁ High
				AE—Right DF LBE—Right DF	75 75	3.08 (0.3587) 3.02 (0.4642)	4.12 (0.5685) 3.66 (0.6224)	0.46 [0.27, 0.65] **	⨁⨁⨁⨁ High
				AE—Left DF LBE—Left DF	75 75	3.05 (0.3991) 3.02 (0.4341)	4.13 (0.5534) 3.26 (0.4452)	0.87 [0.71, 1.03] **	⨁⨁⨁⨁ High
	1RM leg press	Kilogram (higher is better)	Jain & Shinde (2025) India [90]	AE LBE	145 145	57.79 (9.03) 58.34 (10.61)	66.39 (11.23) 59.04 (9.30)	7.35 [4.97, 9.73] **	⨁⨁⨁⨁ High
	5 times sit-to-stand test	Seconds (lower is better)	Khruakhorn & Chiwarakranon (2021) Thailand [91]	AE LBE	17 17	15.36 (2.28) 14.42 (3.71)	11.47 (3.04) 11.20 (3.03)	0.27 [−1.85, 2.39]	⨁⨁⨁◯ Moderate
Knee extensors strength	Dynamometer	Kilograms (higher is better)	Assar et al. (2020) Iran [89]	AE LBE	12 12	14.9 (3.5) 13.4 (1.6)	16.4 (4.3) 16.9 (2.6)	−0.50 [−3.55, 2.55]	⨁⨁◯◯ Low
Functional mobility and balance	Timed up-and-go test	Seconds (lower is better)	Jain & Shinde (2025) India [90]	AE LBE	145 145	12.65 (2.72) 12.48 (2.54)	10.01 (2.57) 11.76 (2.50)	−1.75 [−2.34, −1.16] **	⨁⨁⨁◯ Moderate
		Seconds (lower is better)	Khruakhorn & Chiwarakranon (2021) Thailand [91]	AE LBE	17 17	10.97 (2.73) 10.29 (4.15)	9.68 (2.34) 9.93 (5.09)	−0.25 [−3.06, 2.56]	
Walking speed	40 m fast-paced walk test	Seconds (lower is better)	Jain & Shinde (2025) India [90]	AE LBE	145 145	31.19 (4.21) 30.97 (4.18)	27.06 (2.57) 29.90 (2.67)	−2.84 [−3.45, −2.23] **	⨁⨁⨁⨁ High
Joint position sense	Goniometer	Degrees (lower is better)	Jain & Shinde (2025) India [90]	AE—Left knee LBE—Left knee	145 145	13.14 (2.22) 12.92 (2.50)	10.48 (1.12) 12.61 (1.44)	−2.13 [−2.43, −1.83] **	⨁⨁⨁⨁ High
		Degrees (lower is better)	Jain & Shinde (2025) India [90]	AE—Right knee LBE—Right knee	145 145	12.73 (2.18) 13.11 (2.61)	10.09 (1.37) 12.48 (1.54)	−2.39 [−2.73, −2.05] **	⨁⨁⨁⨁ High
Rectus femoris activity	Electromyography	% of maximum EMG activity (higher indicates greater activation)	Pezeshk et al. (2025) Iran [93]	AE LBE	12 12	0.67 (0.1) 0.66 (0.09)	0.78 (0.09) 0.73 (0.09)	0.05 [−0.03, 0.13]	⨁⨁⨁◯ Moderate
Vastus lateralis activity	Electromyography	% of maximum EMG activity (higher indicates greater activation)	Pezeshk et al. (2025) Iran [93]	AE LBE	12 12	0.68 (0.13) 0.67 (0.07)	0.77 (0.1) 0.75 (0.11)	0.02 [−0.07, 0.11]	⨁⨁⨁◯ Moderate
Biceps femoris activity	Electromyography	% of maximum EMG activity (higher indicates greater activation)	Pezeshk et al. (2025) Iran [93]	AE LBE	12 12	0.55 (0.09) 0.55 (0.08)	0.45 (0.07) 0.50 (0.09)	−0.05 [−0.12, 0.02]	⨁⨁⨁◯ Moderate
Semi-membranosus activity	Electromyography	% of maximum EMG activity (higher indicates greater activation)	Pezeshk et al. (2025) Iran [93]	AE LBE	12 12	0.49 (0.08) 0.52 (0.06)	0.47 (0.06) 0.5 (0.08)	−0.03 [−0.09, 0.03]	⨁⨁⨁◯ Moderate
Overall muscle activity	Electromyography	Net muscle activation % (higher indicates greater overall activation)	Pezeshk et al. (2025) Iran [93]	AE LBE	12 12	3.55 (0.42) 3.54 (0.61)	4.09 (0.13) 4.03 (0.17)	0.06 [−0.07, 0.19]	⨁⨁⨁◯ Moderate
Extensor/flexor co-contraction	Electromyography	Direct co-contraction rate (higher indicates more simultaneous activation of agonist/antagonist muscles)	Pezeshk et al. (2025) Iran [93]	AE LBE	12 12	0.68 (0.07) 0.66 (0.07)	0.65 (0.06) 0.65 (0.07)	0.00 [−0.06, 0.06]	⨁⨁⨁◯ Moderate
Hamstring flexibility	Active knee extension test	Degrees (higher is better)	Shinde & Jain (2025) India [94]	AE—Right LBE—Right	75 75	34.72 (1.68) 36.024 (5.46)	51.05 (0.9986) 37.36 (0.8643)	13.69 [13.39, 13.99] **	⨁⨁⨁⨁ High
				AE—Left LBE—Left	75 75	35.256 (2.013) 35.07 (1.309)	50.01 (0.8563) 35.73 (0.6844)	14.28 [14.03, 14.53] **	⨁⨁⨁⨁ High
Hip joint flexibility	Modified Thomas test	Degrees (higher is better)	Shinde & Jain (2025) India [94]	AE—Right Hip LBE—Right Hip	75 75	107.90 (1.117) 105.37 (0.6529)	112.06 (0.9492) 105.74 (0.8557)	6.32 [6.03, 6.61] **	⨁⨁⨁⨁ High
				AE—Left Hip LBE—Left Hip	75 75	103.37 (0.7671) 105.65 (0.6876)	109.37 (0.6733) 107.01 (0.5325)	2.36 [2.16, 2.56] **	⨁⨁⨁⨁ High
				AE—Right Knee LBE—Right Knee	75 75	110.96 (0.9070) 108.38 (0.7333)	116.61 (0.7515) 110.00 (0.7166)	6.61 [6.37, 6.85] **	⨁⨁⨁⨁ High
				AE—Left Knee LBE—Left Knee	75 75	105.12 (0.7345) 104.82 (0.8116)	113.33 (0.8436) 105.30 (1.052)	8.03 [7.72, 8.34] **	⨁⨁⨁⨁ High
Quadricep amyotrophy	Muscle circumference (difference between the two quadriceps)	Centimetres: lower is better	Slouma et al. (2025) Tunisia [95]	AE LBE	30 30	NR NR	NR NR	NR	NA
Disease-specific severity	Lequesne index	Points: range not reported (lower is better)	Slouma et al. (2025) Tunisia [95]	AE LBE	30 30	10.0 (2.8) 13.6 (4.6)	6.7 (2.1) 12.2 (4.4)	−5.50 [−7.30, −3.70] **	⨁⨁⨁◯ Moderate

AE: aquatic exercise; LBE: land-based exercise; CI: confidence interval; SD: standard deviation; DF: dorsiflexors; HF: hip flexors; HE: hip extensors; KF: knee flexors; KE: knee extensors; PF: plantar flexors; EMG: electromyography; RM: repetition maximum; WOMAC: Western Ontario and McMaster Universities Osteoarthritis Index; NR: not reported; NA: not assessed. * Unadjusted post-intervention mean differences and 95% confidence intervals were calculated by the review authors from the reported group means, standard deviations, and sample sizes. These estimates do not account for baseline differences in the outcome or other participant characteristics and should be interpreted as descriptive between-group comparisons rather than baseline-adjusted treatment effects. § Data were extracted from published graphs. ** Indicate statistically significant between-group differences (95% CI does not include zero). ‡‡ The overall certainty of evidence was evaluated across the five GRADE domains (risk of bias, inconsistency, indirectness, imprecision, and publication bias) and classified as high (⨁⨁⨁⨁), moderate (⨁⨁⨁◯), low (⨁⨁◯◯), or very low (⨁◯◯◯). For outcomes with insufficient information, due to limitations in evaluating the GRADE, their assessment was not performed. Between-group mean differences were always calculated as AE minus LBE. For outcomes where lower scores indicate improvement, negative values favour aquatic exercise and positive values favour land-based exercise. For outcomes where higher scores indicate improvement, positive values favour aquatic exercise and negative values favour land-based exercise.

## Data Availability

No new data were created or analysed in this study. Data sharing is not applicable to this article.

## Supplementary Materials

The following supporting information can be downloaded at: https://www.mdpi.com/article/10.3390/healthcare14203388/s1, Table S1: PRISMA 2020 for Abstracts Checklist; Table S2: PRISMA 2020 Checklist; Table S3: GRADE Summary of Findings.

## Abbreviations

The following abbreviations are used in this manuscript:

1RM	One-Repetition Maximum
5xSTS	Five Times Sit-to-Stand Test
ACR	American College of Rheumatology
AE	Aquatic Exercise
BA	Blinding of Assessors
BMI	Body Mass Index
BS	Blinding of Subjects
BT	Blinding of Therapists
CA	Concealed Allocation
CG	Control Group
CI	Confidence Interval
ECS	Eligibility Criteria Specified
GSB	Groups Similar at Baseline
HRmax	Maximum Heart Rate
ITA	Intention-to-Treat Analysis
KL	Kellgren–Lawrence Radiographic Disease Severity Scale
KOOS	Knee Injury and Osteoarthritis Outcome Score
LBE	Land-Based Exercise
MCID	Minimal Clinically Important Difference
MeSH	Medical Subject Headings
OA	Osteoarthritis
OD	Outcome Data from >85% of Participants
PEDro	Physiotherapy Evidence Database
PEVM	Point Estimates and Variability Measures
PICO(S)	Population, Intervention, Comparison, Outcomes (and Study Design)
PRISMA	Preferred Reporting Items for Systematic Reviews and Meta-Analyses
RA	Random Allocation
RBC	Reported Between-group Comparisons
RCT	Randomised Controlled Trial
RPM	Revolutions Per Minute
SD	Standard Deviation
SE	Standard Error
SF-12	12-item Short Form Health Survey
SF-36	36-item Short Form Health Survey
TRX	Total Resistance Exercise
VAS	Visual Analogue Scale
WHOQOL-BREF	World Health Organization Quality of Life-BREF
WOMAC	Western Ontario and McMaster Universities Osteoarthritis Index
YLD	Years Lived with Disability

## Appendix A. Electronic Search Strategies

### Appendix A.1. Full Search Strategy Used in PubMed

(

(Osteoarthritis, Knee[Mesh] OR Knee Osteoarthritides[Title/Abstract] OR Knee Osteoarthritis[Title/Abstract] OR Knee Osteoartrosis[Title/Abstract] OR Osteoarthritides of Knee[Title/Abstract] OR Osteoarthritis of Knee[Title/Abstract] OR Osteoartrosis of Knee[Title/Abstract] OR Knee OA[Title/Abstract] OR OA of Knee[Title/Abstract] OR Osteoarthritides, Knee[Title/Abstract] OR Osteoarthritis, Knee[Title/Abstract] OR Osteoartrosis, Knee[Title/Abstract] OR Knee, Osteoarthritides of[Title/Abstract] OR Knee, Osteoarthritis of[Title/Abstract] OR Knee, Osteoartrosis of[Title/Abstract] OR OA, Knee[Title/Abstract] OR Knee, OA of[Title/Abstract] OR Gonarthrosis[Title/Abstract])

AND

(Aquatic Therapy[Mesh] OR Hydrotherapy[Mesh] OR Aquatic Therap*[Title/Abstract] OR Pool Therap*[Title/Abstract] OR Ai Chi[Title/Abstract] OR Water Tai Chi[Title/Abstract] OR Aquatic Tai Chi[Title/Abstract] OR Aquatic Exercis*[Title/Abstract] OR Water Exercis*[Title/Abstract] OR Water therap*[Title/Abstract] OR Aquatic Training[Title/Abstract] OR Pool Training[Title/Abstract] OR Water Training[Title/Abstract] OR Aquatic Sport*[Title/Abstract] OR Pool Sport*[Title/Abstract] OR Water Sport*[Title/Abstract] OR Hydrotherap*[Title/Abstract] OR Whirlpool Bath*[Title/Abstract] OR Watsu Therap*[Title/Abstract] OR Water Shiatsu[Title/Abstract] OR Balneotherap*[Title/Abstract] OR Water Run*[Title/Abstract] OR Water Walk*[Title/Abstract] OR Water Aerobic*[Title/Abstract] OR Aquatic-Based[Title/Abstract] OR Pool-Based[Title/Abstract] OR Water-Based[Title/Abstract] OR Aquatic Rehabilitation[Title/Abstract] OR Hydrokinesitherapy[Title/Abstract])

AND

(Exercise[Mesh] OR Resistance Training[Mesh] OR Endurance Training[Mesh] OR High-Intensity Interval Training[Mesh] OR Circuit-Based Exercise[Mesh] OR Exercise Movement Techniques[Mesh] OR Plyometric Exercise[Mesh] OR Physical Conditioning, Human[Mesh] OR Exercis*[Title/Abstract] OR Aerobic*[Title/Abstract] OR Isometric*[Title/Abstract] OR Training[Title/Abstract] OR Physical Activit*[Title/Abstract] OR Resistance*[Title/Abstract] OR Strength*[Title/Abstract] OR Weight Lifting*[Title/Abstract] OR Weight Bearing*[Title/Abstract] OR High Intensity Interval Training[Title/Abstract] OR Circuit-Based[Title/Abstract] OR Circuit Training[Title/Abstract] OR Movement*[Title/Abstract] OR Pilates[Title/Abstract] OR Plyometric*[Title/Abstract] OR Drill*[Title/Abstract] OR Stretch*[Title/Abstract] OR Physical Conditioning[Title/Abstract] OR Land-based[Title/Abstract] OR Functional Training[Title/Abstract] OR Neuromuscular Training[Title/Abstract] OR HIIT[Title/Abstract] OR Balance Training[Title/Abstract] OR Proprioceptive Training[Title/Abstract])

AND

(Pain[Mesh] OR Musculoskeletal Pain[Mesh] OR Physical Suffering*[Title/Abstract] OR Ache*[Title/Abstract] OR Pain*[Title/Abstract] OR Activities of Daily Living[Mesh] OR Daily Living Activit*[Title/Abstract] OR ADL[Title/Abstract] OR Limitation of Activit*[Title/Abstract] OR Physical Function*[Title/Abstract] OR Quality of Life[Mesh] OR Life Quality[Title/Abstract] OR Health-Related Quality Of Life[Title/Abstract] OR Health Related Quality Of Life[Title/Abstract] OR HRQOL[Title/Abstract] OR Health Status[Title/Abstract] OR Visual Analogue Scale[Title/Abstract] OR VAS[Title/Abstract] OR WOMAC[Title/Abstract] OR KOOS[Title/Abstract] OR SF-36[Title/Abstract] OR SF-12[Title/Abstract] OR WHOQOL-BREF[Title/Abstract])

)

### Appendix A.2. Full Search Strategy Used in Web of Science

(

TS = ((Osteoarthritis, Knee OR Knee Osteoarthritides OR Knee Osteoarthritis OR Knee Osteoartrosis OR Osteoarthritides of Knee OR Osteoarthritis of Knee OR Osteoartrosis of Knee OR Knee OA OR OA of Knee OR Osteoarthritides, Knee OR Osteoartrosis, Knee OR Knee, Osteoarthritides of OR Knee, Osteoarthritis of OR Knee, Osteoartrosis of OR OA, Knee OR Knee, OA of OR Gonarthrosis))

AND

TS = ((Aquatic Therap* OR Pool Therap* OR Ai Chi OR Water Tai Chi OR Aquatic Tai Chi OR Aquatic Exercis* OR Water Exercis* OR Water therap* OR Aquatic Training OR Pool Training OR Water Training OR Aquatic Sport* OR Pool Sport* OR Water Sport* OR Hydrotherap* OR Whirlpool Bath* OR Watsu Therap* OR Water Shiatsu OR Balneotherap* OR Water Run* OR Water Walk* OR Water Aerobic* OR Aquatic-Based OR Pool-Based OR Water-Based OR Aquatic Rehabilitation OR Hydrokinesitherapy))

AND

TS = ((Resistance Training OR Endurance Training OR High-Intensity Interval Training OR Circuit-Based Exercise OR Exercise Movement Techniques OR Plyometric Exercise OR Physical Conditioning, Human OR Exercis* OR Aerobic* OR Isometric* OR Training OR Physical Activit* OR Resistance* OR Strength* OR Weight Lifting* OR Weight Bearing* OR High Intensity Interval Training OR Circuit-Based OR Circuit Training OR Movement* OR Pilates OR Plyometric* OR Drill* OR Stretch* OR Physical Conditioning OR Land-based OR Functional Training OR Neuromuscular Training OR HIIT OR Balance Training OR Proprioceptive Training))

AND

TS = ((Musculoskeletal Pain OR Physical Suffering* OR Ache* OR Pain* OR Activities of Daily Living OR Daily Living Activit* OR ADL OR Limitation of Activit* OR Physical Function* OR Quality of Life OR Life Quality OR Health-Related Quality Of Life OR Health Related Quality Of Life OR HRQOL OR Health Status OR Visual Analogue Scale OR VAS OR WOMAC OR KOOS OR SF-36 OR SF-12 OR WHOQOL-BREF))

)

### Appendix A.3. Full Search Strategy Used in Scopus

(

TITLE-ABS-KEY(“Osteoarthritis, Knee” OR “Knee Osteoarthritides” OR “Knee Osteoarthritis” OR “Knee Osteoartrosis” OR “Osteoarthritides of Knee” OR “Osteoarthritis of Knee” OR “Osteoartrosis of Knee” OR “Knee OA” OR “OA of Knee” OR “Osteoarthritides, Knee” OR “Osteoartrosis, Knee” OR “Knee, Osteoarthritides of” OR “Knee, Osteoarthritis of” OR “Knee, Osteoartrosis of” OR “OA, Knee” OR “Knee, OA of” OR “Gonarthrosis”)

AND

TITLE-ABS-KEY(“Aquatic Therap*” OR “Pool Therap*” OR “Ai Chi” OR “Water Tai Chi” OR “Aquatic Tai Chi” OR “Aquatic Exercis*” OR “Water Exercis*” OR “Water therap*” OR “Aquatic Training” OR “Pool Training” OR “Water Training” OR “Aquatic Sport*” OR “Pool Sport*” OR “Water Sport*” OR “Hydrotherap*” OR “Whirlpool Bath*” OR “Watsu Therap*” OR “Water Shiatsu” OR “Balneotherap*” OR “Water Run*” OR “Water Walk*” OR “Water Aerobic*” OR “Aquatic-Based” OR “Pool-Based” OR “Water-Based” OR “Aquatic Rehabilitation” OR “Hydrokinesitherapy”)

AND

TITLE-ABS-KEY(“Resistance Training” OR “Endurance Training” OR “High-Intensity Interval Training” OR “Circuit-Based Exercise” OR “Exercise Movement Techniques” OR “Plyometric Exercise” OR “Physical Conditioning, Human” OR “Exercis*” OR “Aerobic*” OR “Isometric*” OR “Training” OR “Physical Activit*” OR “Resistance*” OR “Strength*” OR “Weight Lifting*” OR “Weight Bearing*” OR “High Intensity Interval Training” OR “Circuit-Based” OR “Circuit Training” OR “Movement*” OR “Pilates” OR “Plyometric*” OR “Drill*” OR “Stretch*” OR “Physical Conditioning” OR “Land-based” OR “Functional Training” OR “Neuromuscular Training” OR “HIIT” OR “Balance Training” OR “Proprioceptive Training”)

AND

TITLE-ABS-KEY(“Musculoskeletal Pain” OR “Physical Suffering*” OR “Ache*” OR “Pain*” OR “Activities of Daily Living” OR “Daily Living Activit*” OR “ADL” OR “Limitation of Activit*” OR “Physical Function*” OR “Quality of Life” OR “Life Quality” OR “Health-Related Quality Of Life” OR “Health Related Quality Of Life” OR “HRQOL” OR “Health Status” OR “Visual Analogue Scale” OR “VAS” OR “WOMAC” OR “KOOS” OR “SF-36” OR “SF-12” OR “WHOQOL-BREF”)

### Appendix A.4. Full Search Strategy Used in PEDro

Abstract & Title: Osteoarthr* Aquatic, Osteoarthr* Water, Osteoarthr* Pool

Body part: lower leg or knee

Method: clinical trial

Match all search terms (AND)
