# Supplementary material for: Comparative Effectiveness of Aquatic Versus Land-Based Exercise on Pain, Physical Function, and Quality of Life in People with Knee Osteoarthritis: An Updated Systematic Review of Randomised Controlled Trials
Source: Healthcare (Basel). 2026 Oct 11;14(20):3388. doi: 10.3390/healthcare14203388 (PMC13704367; doi:10.3390/healthcare14203388)
Supplement: Supplementary file 1 [file healthcare-14-03388-s001.zip › healthcare-4436632-supplementary.pdf]

Table S1 - PRISMA 2020 for Abstracts Checklist

Section and Topic	Item #	Checklist item	Reported (Yes/No)
**TITLE**			
Title	1	Identify the report as a systematic review.	Yes
**BACKGROUND**			
Objectives	2	Provide an explicit statement of the main objective(s) or question(s) the review addresses.	Yes
**METHODS**			
Eligibility criteria	3	Specify the inclusion and exclusion criteria for the review.	Partially
Information sources	4	Specify the information sources (e.g. databases, registers) used to identify studies and the date when each was last searched.	Partially
Risk of bias	5	Specify the methods used to assess risk of bias in the included studies.	Yes
Synthesis of results	6	Specify the methods used to present and synthesise results.	Yes
**RESULTS**			
Included studies	7	Give the total number of included studies and participants and summarise relevant characteristics of studies.	Yes
Synthesis of results	8	Present results for main outcomes, preferably indicating the number of included studies and participants for each. If meta-analysis was done, report the summary estimate and confidence/credible interval. If comparing groups, indicate the direction of the effect (i.e. which group is favoured).	Yes
**DISCUSSION**			
Limitations of evidence	9	Provide a brief summary of the limitations of the evidence included in the review (e.g. study risk of bias, inconsistency and imprecision).	Yes
Interpretation	10	Provide a general interpretation of the results and important implications.	Yes
**OTHER**			
Funding	11	Specify the primary source of funding for the review.	No
Registration	12	Provide the register name and registration number.	No

From: Page MJ, McKenzie JE, Bossuyt PM, Boutron I, Hoffmann TC, Mulrow CD, et al. The PRISMA 2020 statement: an updated guideline for reporting systematic reviews. BMJ 2021;372:n71. doi: 10.1136/bmj.n71. This work is licensed under CC BY 4.0. To view a copy of this license, visit https://creativecommons.org/licenses/by/4.0/

Table S2 - PRISMA 2020 Checklist

Section and Topic	Item #	Checklist item	Location where item is reported
**TITLE**			
Title	1	Identify the report as a systematic review.	Title page: article type “Systematic Review” and manuscript title.
**ABSTRACT**			
Abstract	2	See the PRISMA 2020 for Abstracts checklist.	Abstract; Table S1: PRISMA 2020 for Abstracts Checklist.
**INTRODUCTION**			
Rationale	3	Describe the rationale for the review in the context of existing knowledge.	Section 1: Introduction.
Objectives	4	Provide an explicit statement of the objective(s) or question(s) the review addresses.	Section 1: Introduction, final paragraph; the objective specifies the update period from 25 September 2018 to 31 July 2025.
**METHODS**			
Eligibility criteria	5	Specify the inclusion and exclusion criteria for the review and how studies were grouped for the syntheses.	Sections 2.2, 2.3 and 2.5: eligibility criteria and outcome domains; Section 2.7: grouping by outcome domain and measurement instrument and descriptive classification of intervention comparability.
Information sources	6	Specify all databases, registers, websites, organisations, reference lists and other sources searched or consulted to identify studies. Specify the date when each source was last searched or consulted.	Section 2.1: PubMed, Web of Science, Scopus, PEDro and citation searching; electronic searches to 31 July 2025.
Search strategy	7	Present the full search strategies for all databases, registers and websites, including any filters and limits used.	Appendix A: A.1 PubMed; A.2 Web of Science; A.3 Scopus; A.4 PEDro.
Selection process	8	Specify the methods used to decide whether a study met the inclusion criteria of the review, including how many reviewers screened each record and each report retrieved, whether they worked independently, and if applicable, details of automation tools used in the process.	Section 2.4: titles/abstracts and full texts assessed independently by two reviewers, with disagreements resolved with a third reviewer; no translation tools were required. Figure 1 presents the selection process.
Data collection process	9	Specify the methods used to collect data from reports, including how many reviewers collected data from each report, whether they worked independently, any processes for obtaining or confirming data from study investigators, and if applicable, details of automation tools used in the process.	Section 2.4: data independently extracted by two reviewers, with disagreements resolved through discussion and, when

Table S2 - PRISMA 2020 Checklist

Section and Topic	Item #	Checklist item	Location where item is reported
			necessary, a third reviewer; authors of seven RCTs were contacted regarding missing or unclear data.
Data items	10a	List and define all outcomes for which data were sought. Specify whether all results that were compatible with each outcome domain in each study were sought (e.g. for all measures, time points, analyses), and if not, the methods used to decide which results to collect.	Sections 2.3 and 2.4: primary and secondary outcomes; pre- and post-intervention values, group-specific sample sizes and outcome data were sought. Section 2.7 describes the quantitative information used in the synthesis.
	10b	List and define all other variables for which data were sought (e.g. participant and intervention characteristics, funding sources). Describe any assumptions made about any missing or unclear information.	Section 2.4: study, participant, diagnostic, disease-severity and intervention characteristics; missing or unclear information recorded as "not reported"; Tables 2 and 3.
Study risk of bias assessment	11	Specify the methods used to assess risk of bias in the included studies, including details of the tool(s) used, how many reviewers assessed each study and whether they worked independently, and if applicable, details of automation tools used in the process.	Section 2.6 and Table 1: methodological quality assessed using individual PEDro items by two independent reviewers. Section 2.8 and Supplementary Table S3: structured outcome-level risk-of-bias judgements for GRADE.
Effect measures	12	Specify for each outcome the effect measure(s) (e.g. risk ratio, mean difference) used in the synthesis or presentation of results.	Sections 2.4 and 2.7: unadjusted post-intervention between-group mean differences with 95% CIs; Tables 4 and 5.
Synthesis methods	13a	Describe the processes used to decide which studies were eligible for each synthesis (e.g. tabulating the study intervention characteristics and comparing against the planned groups for each synthesis (item #5)).	Sections 2.2, 2.3, 2.5 and 2.7: PICOS, predefined outcome domains, eligibility criteria, grouping by outcome domain and measurement instrument, and intervention-comparability classification.
	13b	Describe any methods required to prepare the data for presentation or synthesis, such as handling of missing summary statistics, or data conversions.	Section 2.7: SD estimation from SE, graph digitisation, mean/SD estimation from median/minimum/maximum, and calculation of mean

Table S2 - PRISMA 2020 Checklist

Section and Topic	Item #	Checklist item	Location where item is reported
			differences and 95% CIs.
	13c	Describe any methods used to tabulate or visually display results of individual studies and syntheses.	Section 2.7; Tables 1–5 present study characteristics, methodological quality, intervention protocols and outcome results; Figure 1 presents study selection.
	13d	Describe any methods used to synthesize results and provide a rationale for the choice(s). If meta-analysis was performed, describe the model(s), method(s) to identify the presence and extent of statistical heterogeneity, and software package(s) used.	Section 2.7: structured synthesis without meta-analysis, with rationale based on the limited number of comparable studies, clinical and methodological heterogeneity, intervention comparability, reporting differences and correlated outcomes; SWiM guidance followed.
	13e	Describe any methods used to explore possible causes of heterogeneity among study results (e.g. subgroup analysis, meta-regression).	No formal subgroup analysis or meta-regression was conducted. Section 2.7 explains why formal heterogeneity analyses were not appropriate; possible sources of heterogeneity are considered descriptively in Sections 3.3–3.5 and Section 4.
	13f	Describe any sensitivity analyses conducted to assess robustness of the synthesized results.	No sensitivity analyses were conducted; the rationale is stated in Section 2.7.
Reporting bias assessment	14	Describe any methods used to assess risk of bias due to missing results in a synthesis (arising from reporting biases).	Section 2.8: publication bias considered within the GRADE framework. Limitations in assessing publication bias, including the absence of grey-literature and trial-registry searches, are acknowledged in Section 4 and Supplementary Table S3.
Certainty assessment	15	Describe any methods used to assess certainty (or confidence) in the body of evidence for an outcome.	Section 2.8: GRADE assessment across risk of bias, inconsistency, indirectness, imprecision and publication bias; Supplementary Table S3 presents domain-level

Table S2 - PRISMA 2020 Checklist

Section and Topic	Item #	Checklist item	Location where item is reported
			judgements and reasons for downgrading.
**RESULTS**			
Study selection	16a	Describe the results of the search and selection process, from the number of records identified in the search to the number of studies included in the review, ideally using a flow diagram.	Section 3.1 and Figure 1: records identified, duplicate and date-based removals, screening, retrieval, eligibility assessment, exclusions and included studies.
	16b	Cite studies that might appear to meet the inclusion criteria, but which were excluded, and explain why they were excluded.	Section 3.1 and Figure 1: three full-text reports were excluded and the reasons are reported.
Study characteristics	17	Cite each included study and present its characteristics.	Section 3.3; Tables 2 and 3.
Risk of bias in studies	18	Present assessments of risk of bias for each included study.	Section 3.2 and Table 1: study-level PEDro item assessments. Section 3.6 and Supplementary Table S3: outcome-level risk-of-bias judgements used in GRADE.
Results of individual studies	19	For all outcomes, present, for each study: (a) summary statistics for each group (where appropriate) and (b) an effect estimate and its precision (e.g. confidence/credible interval), ideally using structured tables or plots.	Tables 4 and 5: group-specific pre- and post-intervention summary statistics, unadjusted post-intervention between-group mean differences and 95% CIs.
Results of syntheses	20a	For each synthesis, briefly summarise the characteristics and risk of bias among contributing studies.	Sections 3.2–3.5 and Tables 1–5 summarise contributing studies, methodological limitations, intervention comparability and outcome characteristics; Supplementary Table S3 presents outcome-level GRADE judgements.
	20b	Present results of all statistical syntheses conducted. If meta-analysis was done, present for each the summary estimate and its precision (e.g. confidence/credible interval) and measures of statistical heterogeneity. If comparing groups, describe the direction of the effect.	Sections 3.4 and 3.5; Tables 4 and 5. No meta-analysis was conducted; unadjusted post-intervention between-group estimates, 95% CIs and direction of effect are presented descriptively.
	20c	Present results of all investigations of possible causes of heterogeneity among study results.	No formal investigation of heterogeneity was

Table S2 - PRISMA 2020 Checklist

Section and Topic	Item #	Checklist item	Location where item is reported
			conducted; differences across studies, interventions, instruments and results are described in Sections 3.3–3.5 and Section 4.
	20d	Present results of all sensitivity analyses conducted to assess the robustness of the synthesized results.	No sensitivity analyses were conducted; the rationale is stated in Section 2.7.
Reporting biases	21	Present assessments of risk of bias due to missing results (arising from reporting biases) for each synthesis assessed.	Publication bias is reported as a GRADE domain in Supplementary Table S3. The limited ability to assess publication bias is acknowledged in Sections 3.6 and 4.
Certainty of evidence	22	Present assessments of certainty (or confidence) in the body of evidence for each outcome assessed.	Section 3.6; Tables 4 and 5; Supplementary Table S3 presents the five GRADE domains, reasons for downgrading and overall certainty for each assessed outcome.
**DISCUSSION**			
Discussion	23a	Provide a general interpretation of the results in the context of other evidence.	Section 4: Discussion.
	23b	Discuss any limitations of the evidence included in the review.	Section 4: limitations related to risk of bias, baseline imbalance, unadjusted estimates, intervention heterogeneity, imprecision, correlated outcomes, adherence, safety reporting and short follow-up.
	23c	Discuss any limitations of the review processes used.	Section 4: limitations arising from exclusion of grey literature and trial registries, language and outcome-instrument restrictions, the temporal scope, unavailable adjusted estimates, inability to pool results or conduct sensitivity analyses, and unavailable pre-consensus agreement data.
	23d	Discuss implications of the results for practice, policy, and future research.	Sections 4 and 5: implications for individualised exercise selection, interpretation of

Table S2 - PRISMA 2020 Checklist

Section and Topic	Item #	Checklist item	Location where item is reported
			comparative effects and priorities for future research.
**OTHER INFORMATION**			
Registration and protocol	24a	Provide registration information for the review, including register name and registration number, or state that the review was not registered.	Section 2.1: PROSPERO registration CRD420251143959.
	24b	Indicate where the review protocol can be accessed, or state that a protocol was not prepared.	Section 2.1: PROSPERO registration CRD420251143959; the registered protocol is accessible through the PROSPERO record.
	24c	Describe and explain any amendments to information provided at registration or in the protocol.	Section 2.1 states that the registered methods and temporal scope were applied consistently; no protocol amendments are reported.
Support	25	Describe sources of financial or non-financial support for the review, and the role of the funders or sponsors in the review.	Funding statement: "This research received no external funding"; Acknowledgments.
Competing interests	26	Declare any competing interests of review authors.	Conflicts of Interest statement.
Availability of data, code and other materials	27	Report which of the following are publicly available and where they can be found: template data collection forms; data extracted from included studies; data used for all analyses; analytic code; any other materials used in the review.	Data Availability Statement; extracted study and outcome data are presented in Tables 2–5 and Supplementary Table S3; full search strategies are provided in Appendix A. Data-collection forms and analytic code are not reported as publicly available.

From: Page MJ, McKenzie JE, Bossuyt PM, Boutron I, Hoffmann TC, Mulrow CD, et al. The PRISMA 2020 statement: an updated guideline for reporting systematic reviews. BMJ 2021;372:n71. doi: 10.1136/bmj.n71.
This work is licensed under CC BY 4.0. To view a copy of this license, visit https://creativecommons.org/licenses/by/4.0/

Table S3 - GRADE Summary of Findings

Certainty assessment							
**Outcome**	**Participants (studies)**	**Risk of Bias**	**Inconsistency**	**Indirectness**	**Imprecision**	**Publication Bias**	**Overall certainty of evidence**
**VAS rest**	500 (3 RCTs)	Serious	Not serious	Not serious	Serious	Undetected	⊕⊕◯◯ Low[a,b]
**VAS activity**	524 (4 RCTs)	Serious	Serious	Not serious	Serious	Undetected	⊕◯◯◯ Very low[b,c,d]
**WOMAC pain**	110 (3 RCTs)	Serious	Very serious	Not serious	Very serious	Undetected	⊕◯◯◯ Very low[a,e,f]
**WOMAC physical function**	110 (3 RCTs)	Serious	Very serious	Not serious	Very serious	Undetected	⊕◯◯◯ Very low[a,d,f]
**WOMAC total**	534 (4 RCTs)	Serious	Very serious	Not serious	Very serious	Undetected	⊕◯◯◯ Very low[c,d,f]
**SF-36 physical functioning**	199 (3 RCTs)	Serious	Very serious	Not serious	Very serious	Undetected	⊕◯◯◯ Very low[a,f,g]
**SF-36 role-physical**	199 (3 RCTs)	Serious	Very serious	Not serious	Very serious	Undetected	⊕◯◯◯ Very low[a,f,g]
**SF-36 bodily pain**	199 (3 RCTs)	Serious	Very serious	Not serious	Very serious	Undetected	⊕◯◯◯ Very low[a,f,g]
**SF-36 general health**	199 (3 RCTs)	Serious	Very serious	Not serious	Very serious	Undetected	⊕◯◯◯ Very low[a,f,g]
**SF-36 vitality**	199 (3 RCTs)	Serious	Very serious	Not serious	Very serious	Undetected	⊕◯◯◯ Very low[a,b,g]
**SF-36 social functioning**	199 (3 RCTs)	Serious	Very serious	Not serious	Very serious	Undetected	⊕◯◯◯ Very low[a,f,g]

Certainty assessment							
**SF-36 mental health**	199<br>(3 RCTs)	Serious	Very serious	Not serious	Very serious	Undetected	⊕◯◯◯<br>Very low[a,f,g]
**Functional - 6-minute walk test**	46<br>(2 RCTs)	Serious	Very serious	Not serious	Very serious	Undetected	⊕◯◯◯<br>Very low[c,f,g]
**Functional - Stair Test**	50<br>(2 RCTs)	Not serious	Not serious	Serious	Serious	Undetected	⊕⊕◯◯<br>Low[b,h]
**Functional - Timed up and Go**	324<br>(2 RCTs)	Not serious	Not serious	Not serious	Serious	Undetected	⊕⊕⊕◯<br>Moderate[b]
**Stiffness - WOMAC**	134<br>(4 RCTs)	Serious	Very serious	Not serious	Serious	Undetected	⊕◯◯◯<br>Very low[b,c,g]
**VAS unspecified**	30<br>(1 RCT)	Not serious	Not serious	Not serious	Serious	Undetected	⊕⊕⊕◯<br>Moderate[g,i]
**KOOS pain**	34<br>(1 RCT)	Serious	Not serious	Not serious	Very serious	Undetected	⊕◯◯◯<br>Very low[c,f,i]
**WHOQOL-BREF physical**	34<br>(1 RCT)	Not serious	Not serious	Not serious	Very serious	Undetected	⊕⊕◯◯<br>Low[g,i]
**WHOQOL-BREF mental**	34<br>(1 RCT)	Not serious	Not serious	Not serious	Very serious	Undetected	⊕⊕◯◯<br>Low[g,i]
**WHOQOL-BREF social**	34<br>(1 RCT)	Not serious	Not serious	Not serious	Very serious	Undetected	⊕⊕◯◯<br>Low[g,i]
**WHOQOL-BREF environment**	34<br>(1 RCT)	Not serious	Not serious	Not serious	Very serious	Undetected	⊕⊕◯◯<br>Low[g]
**WHOQOL-BREF total**	34<br>(1 RCT)	Not serious	Not serious	Not serious	Very serious	Undetected	⊕⊕◯◯<br>Low[g,i]
**SF-12 physical domain**	60<br>(1 RCT)	Not serious	Not serious	Not serious	Not serious	Undetected	⊕⊕⊕⊕<br>High[i]

Certainty assessment							
**SF-12 mental domain**	60 (1 RCT)	Not serious	Not serious	Not serious	Serious	Undetected	⊕⊕⊕◯ Moderate[b,i]
**Maximum oxygen consumption**	16 (1 RCT)	Not serious	Not serious	Not serious	Very serious	Undetected	⊕⊕◯◯ Low[g,i]
**Anaerobic threshold**	16 (1 RCT)	Not serious	Not serious	Not serious	Very serious	Undetected	⊕⊕◯◯ Low[g,i]
**Berg balance scale**	24 (1 RCT)	Not serious	Not serious	Not serious	Very serious	Undetected	⊕⊕◯◯ Low[g,i]
**Felson's questionnaire**	24 (1 RCT)	Not serious	Not serious	Not serious	Serious	Undetected	⊕⊕⊕◯ Moderate[b,i]
**Bubble inclinometer**	24 (1 RCT)	Not serious	Not serious	Not serious	Very serious	Undetected	⊕⊕◯◯ Low[g,i]
**2D video-based kinematics**	24 (1 RCT)	Not serious	Not serious	Not serious	Serious	Undetected	⊕⊕⊕◯ Moderate[b,i]
**Goniometer - Range of Motion**	60 (1 RCT)	Not serious	Not serious	Not serious	Serious	Undetected	⊕⊕⊕◯ Moderate[b,i]
**Manual muscle testing - right hip flexors**	150 (1 RCT)	Not serious	Not serious	Not serious	Not serious	Undetected	⊕⊕⊕⊕ High[i]
**Manual muscle testing - left hip flexors**	150 (1 RCT)	Not serious	Not serious	Not serious	Not serious	Undetected	⊕⊕⊕⊕ High[i]
**Manual muscle testing - right hip extensors**	150 (1 RCT)	Not serious	Not serious	Not serious	Not serious	Undetected	⊕⊕⊕⊕ High[i]
**Manual muscle testing - left hip extensors**	150 (1 RCT)	Not serious	Not serious	Not serious	Not serious	Undetected	⊕⊕⊕⊕ High[i]
**Manual muscle testing - right knee flexors**	150 (1 RCT)	Not serious	Not serious	Not serious	Not serious	Undetected	⊕⊕⊕⊕ High[i]

Certainty assessment							
**Manual muscle testing - left knee flexors**	150 (1 RCT)	Not serious	Not serious	Not serious	Not serious	Undetected	⊕⊕⊕⊕ High[i]
**Manual muscle testing - right knee extensors**	150 (1 RCT)	Not serious	Not serious	Not serious	Not serious	Undetected	⊕⊕⊕⊕ High[i]
**Manual muscle testing - left knee extensors**	150 (1 RCT)	Not serious	Not serious	Not serious	Not serious	Undetected	⊕⊕⊕⊕ High[i]
**Manual muscle testing - right plantar flexors**	150 (1 RCT)	Not serious	Not serious	Not serious	Not serious	Undetected	⊕⊕⊕⊕ High[i]
**Manual muscle testing - left plantar flexors**	150 (1 RCT)	Not serious	Not serious	Not serious	Not serious	Undetected	⊕⊕⊕⊕ High[i]
**Manual muscle testing - right dorsiflexors**	150 (1 RCT)	Not serious	Not serious	Not serious	Not serious	Undetected	⊕⊕⊕⊕ High[i]
**Manual muscle testing - left dorsiflexors**	150 (1 RCT)	Not serious	Not serious	Not serious	Not serious	Undetected	⊕⊕⊕⊕ High[i]
**1-RM leg press**	290 (1 RCT)	Not serious	Not serious	Not serious	Not serious	Undetected	⊕⊕⊕⊕ High[i]
**5 times sit-to-stand**	34 (1 RCT)	Not serious	Not serious	Not serious	Serious	Undetected	⊕⊕⊕◯ Moderate[b,i]
**Dynamometer - knee extensor strength**	24 (1 RCT)	Not serious	Not serious	Not serious	Very serious	Undetected	⊕⊕◯◯ Low[g,i]
**40-meter fast-paced walk test**	290 (1 RCT)	Not serious	Not serious	Not serious	Not serious	Undetected	⊕⊕⊕⊕ High[i]
**Goniometer - joint position sense - left knee**	290 (1 RCT)	Not serious	Not serious	Not serious	Not serious	Undetected	⊕⊕⊕⊕ High[i]
**Goniometer - joint position sense - right knee**	290 (1 RCT)	Not serious	Not serious	Not serious	Not serious	Undetected	⊕⊕⊕⊕ High[i]

Certainty assessment							
**Electromyography - rectus femoris**	24 (1 RCT)	Not serious	Not serious	Not serious	Serious	Undetected	⊕⊕⊕◯ Moderate[g,i]
**Electromyography - vastus lateralis**	24 (1 RCT)	Not serious	Not serious	Not serious	Serious	Undetected	⊕⊕⊕◯ Moderate[g,i]
**Electromyography - biceps femoris**	24 (1 RCT)	Not serious	Not serious	Not serious	Serious	Undetected	⊕⊕⊕◯ Moderate[g,i]
**Electromyography - semimembranosus**	24 (1 RCT)	Not serious	Not serious	Not serious	Serious	Undetected	⊕⊕⊕◯ Moderate[g,i]
**Electromyography - overall muscle activity**	24 (1 RCT)	Not serious	Not serious	Not serious	Serious	Undetected	⊕⊕⊕◯ Moderate[g,i]
**Electromyography - extensor/flexor co-contraction**	24 (1 RCT)	Not serious	Not serious	Not serious	Serious	Undetected	⊕⊕⊕◯ Moderate[g,i]
**Active knee extension test - right**	150 (1 RCT)	Not serious	Not serious	Not serious	Not serious	Undetected	⊕⊕⊕⊕ High[i]
**Active knee extension test - left**	150 (1 RCT)	Not serious	Not serious	Not serious	Not serious	Undetected	⊕⊕⊕⊕ High[i]
**Modified Thomas test - right hip**	150 (1 RCT)	Not serious	Not serious	Not serious	Not serious	Undetected	⊕⊕⊕⊕ High[i]
**Modified Thomas test - left hip**	150 (1 RCT)	Not serious	Not serious	Not serious	Not serious	Undetected	⊕⊕⊕⊕ High[i]
**Modified Thomas test - right knee**	150 (1 RCT)	Not serious	Not serious	Not serious	Not serious	Undetected	⊕⊕⊕⊕ High[i]
**Modified Thomas test - left knee**	150 (1 RCT)	Not serious	Not serious	Not serious	Not serious	Undetected	⊕⊕⊕⊕ High[i]
**Lequesne Index**	60 (1 RCT)	Not serious	Not serious	Not serious	Serious	Undetected	⊕⊕⊕◯ Moderate[b,i]

Explanations

a. Several contributing studies had methodological limitations, including inadequate reporting or absence of allocation concealment, participant and therapist blinding, assessor blinding, and/or complete outcome data, resulting in important concerns about risk of bias.

b. The 95% confidence interval was sufficiently wide to include effects compatible with little or no between-group difference and potentially clinically important effects, indicating imprecision in the estimated treatment effect.

c. Several contributing studies had methodological limitations, although the overall concerns were less extensive than for outcomes downgraded for more serious risk of bias.

d. Effect estimates varied substantially across studies, with differences in the magnitude and/or direction of effects that could not be adequately explained by differences in intervention characteristics, populations, or outcome measurement.

e. At least one contributing study reported an effect estimate substantially different from those of the other studies, contributing to unexplained variability in the magnitude and/or direction of effects.

f. The 95% confidence intervals were very wide and included effects favouring aquatic exercise, little or no difference, and/or effects favouring land-based exercise, resulting in substantial uncertainty regarding the magnitude and direction of the effect.

g. The direction of effect varied across studies, with estimates favouring aquatic exercise in some studies and land-based exercise or little/no difference in others, and this variability was not adequately explained by differences across studies.

h. The outcome was assessed using modified testing protocols, including differences in test characteristics such as step height, which may limit comparability of effect estimates across studies.

i. Only one study contributed to this outcome, therefore, inconsistency across studies could not be assessed, and the limited number of studies also prevented meaningful assessment of publication bias. No downgrade was applied solely because these domains could not be assessed.
